# An Efficient Thiol‐ene Mediated Protocol for Thiolated Peptide Synthesis and On‐Resin Diversification

**DOI:** 10.1002/chem.202501372

**Published:** 2025-05-23

**Authors:** Nikita Ostrovitsa, Conor Williams, Konstantin Raabe, Joshua T. McLean, Markus Muttenthaler, Eoin M. Scanlan

**Affiliations:** ^1^ School of Chemistry Trinity College Dublin College Green Dublin 2 Ireland; ^2^ Institute of Biological Chemistry University of Vienna Währingerstrabe 38 Vienna 1090 Austria; ^3^ Institute for Molecular Bioscience The University of Queensland Brisbane 4072 Australia

**Keywords:** disulfide, ligation, peptide, solid‐phase, thiol‐ene

## Abstract

The unique nucleophilic and redox properties of the sulfhydryl group render it highly useful as a synthetic handle for the diversification of peptide structure, including macrocyclization, ligation, and bioconjugation. Herein, a sequential acyl‐thiol‐ene/*S*‐deacetylation protocol for selectively installing thiol residues onto bioactive peptides on‐resin is demonstrated. Through judicious placement of appropriate unsaturated residues, the hydrothiolation/*S*‐deacetylation protocol offers a novel synthetic strategy to investigate the structure‐activity relationship of disulfide‐containing peptides displaying different ring sizes. Furthermore, a new and generally applicable fluorescent labeling strategy is introduced to facilitate direct on‐resin conjugation without intermediate purification steps. These new methods provide a robust and versatile platform for peptide macrocyclization and bioconjugation, with broad applications in peptide synthesis and chemical biology.

## Graphical abstract

Herein, a sequential acyl‐thiol‐ene/*S*‐deacetylation protocol for selective installation of thiol residues onto synthetic peptides, on‐resin, is demonstrated. The hydrothiolation/S‐deacetylation protocol offers a novel synthetic tool for direct peptide conjugation on‐resin and for the preparation of novel disulfide containing neuropeptides displaying non‐natural ring sizes without intermediate purification steps.

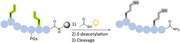

## 1 Introduction

Driven by recent advances in library screening and *in silico* methods, peptide therapeutics continue to emerge as promising clinical candidates for treating a diverse range of diseases.^[^ ^1^, ^2^, ^3^, ^4^ ^]^ Peptide drugs occupy a unique chemical space between traditional small‐molecule therapeutics and larger biologics, offering scope to investigate previously undruggable targets, including protein‐protein interactions.^[^ ^1^, ^5^ ^]^ They possess many desirable characteristics of drug candidates, including high target affinity and specificity, well‐defined mechanisms of action, and low toxicity.^[^ ^6^, ^7^, ^8^ ^]^ Developing synthetic methodologies for peptide diversification and cyclization, particularly strategies compatible with on‐resin synthesis, is an area of intensive research. Common reactions employed for late‐stage chemical modification of peptides include disulfide‐bond formations,^[^ ^9^ ^]^ ring‐closing metathesis (RCM),^[^ ^10^ ^]^ and various “click” reactions, including the well‐established, azide‐alkyne cycloaddition.^[^ ^11^, ^12^ ^]^

The radical‐mediated thiol‐ene reaction has been extensively investigated for applications in peptide synthesis, including macrocyclization, stapling, bioconjugation, and surface modification, and has recently been reviewed in this context by us and others.^[^ ^13^, ^14^ ^]^ The process involves anti‐Markovnikov hydrothiolation of an olefin, furnishing a robust thioether or thioester product. The reaction follows a radical chain mechanism, initiated either thermally or photochemically upon homolytic cleavage of the labile S‐H bond.^[^ ^13^ ^]^ The thiol‐ene reaction is often classified as a “click” reaction due to its high efficiency, atom economy, and mild reaction conditions.^[^ ^14^ ^]^ Furthermore, it is compatible with a broad range of functional groups and solvent systems, including aqueous conditions, eliminating the need for complex protecting groups and thereby streamlining biomolecular synthesis.^[^ ^15^ ^]^ Consequently, the thiol‐ene reaction has been widely utilized across multiple disciplines, including polymer chemistry,^[^ ^16^ ^]^ surface chemistry,^[^ ^17^ ^]^ carbohydrate chemistry,^[^ ^18^ ^]^ and chemical biology.^[^ ^19^ ^]^ This reaction has been extensively studied within our group, particularly for applications in peptide ligation and thiolactonization toward drug discovery.^[^ ^20^, ^21^, ^22^ ^]^

**Scheme 1:**
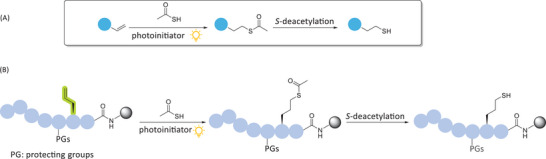
A) ATE and *S*‐deacetylation sequence used to synthesize thiol derivatives. B) Application of sequential ATE and *S*‐deacetylation for direct on‐resin peptide hydrothiolation.

Although thiol‐ene‐mediated reactions have been utilized for various peptide modifications, their potential for direct hydrothiolation of peptide substrates on resin remains underexplored. The radical‐mediated acyl‐thiol‐ene (ATE) reaction between thioacetic acid and alkenes, followed by *S*‐deacetylation of the thioacetate intermediate to furnish a thiol residue (Scheme 1A), has been widely employed in the synthesis of thiol‐containing substrates, including linkers for gold nanoparticles,^[^ ^23^ ^]^ gold surfaces,^[^ ^24^ ^]^ and fullerenes^[^ ^25^ ^]^ as well as thiolated biomolecules including sugars^[^ ^26^ ^]^ and steroids.^[^ ^27^ ^]^ However, the application of direct peptide hydrothiolation on resin has not been investigated, despite its synthetic scope for late‐stage peptide diversification and drug discovery. Herein, we present, for the first time, a general synthetic protocol for regioselective hydrothiolation of unsaturated peptide residues on resin via a sequential ATE/*S*‐deacetylation protocol. Incorporating commercially available unsaturated amino acids during solid‐phase peptide synthesis (SPPS) enables regioselective insertion of a thiol residue under mild conditions prior to peptide cleavage and global deprotection (Scheme 1B). Following careful optimization of the two on‐resin reactions, the utility of this new methodology is demonstrated through the generation of a small library of disulfide‐based cyclic neuropeptide analogs of vasopressin (VP) and somatostatin (SST). Systematic replacement of the cysteine residues with longer alkenyl amino acids, such as allylglycine (Allyl‐Gly), followed by on‐resin hydrothiolation, *S*‐deacetylation, and peptide cyclization via disulfide bond formation in solution, enabled access to novel VP and SST derivatives with increased ring sizes. As a proof‐of‐concept, this methodology was applied to the on‐resin thiol insertion followed by fluorophore labeling of a linear arginyl‐glycyl‐aspartic acid (RGD)‐containing peptide, commonly used in cancer cell targeting.^[^ ^28^ ^]^

## 2 Results and Discussion

### 2.1 On‐Resin Optimization

**Figure 1:**
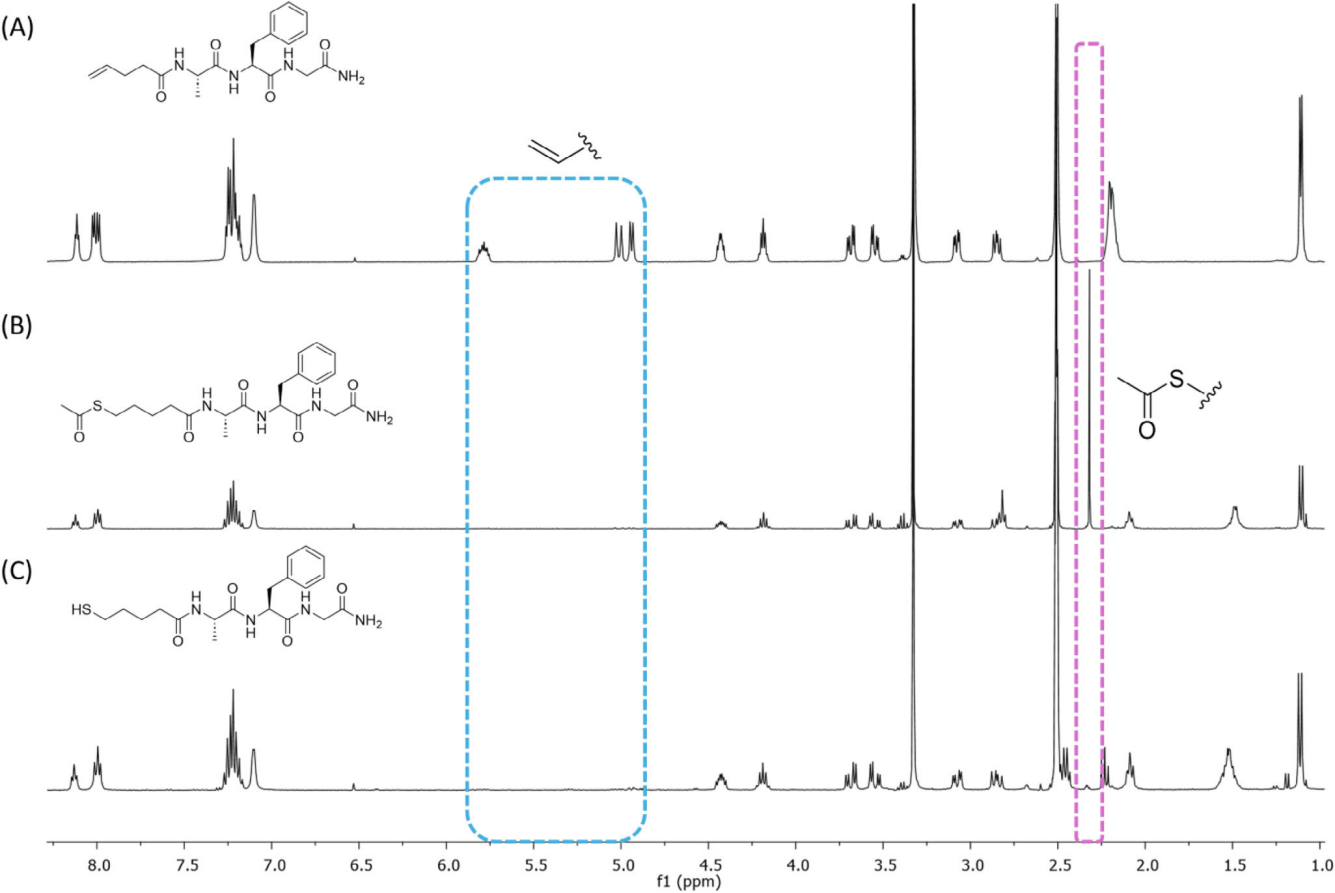
A) ^1^H NMR spectrum of the starting material 1. B) ^1^H NMR spectrum of the intermediate thioacetate 2 under the optimized conditions (Table 1, entry 3) illustrating the disappearance of the distinct alkene peaks of 1 and the appearance of the new singlet (CH_3_CO‐) peak. C) ^1^H NMR spectrum of the final free thiol peptide 4 under the optimized conditions (Table 2, entry 6) illustrating the disappearance of the distinct singlet (CH_3_CO‐) peak of 2.

Initial optimization studies were performed using the 4‐pentenoic acid‐capped tripeptide **1**, assembled on Rink amide resin. The thiol‐ene reaction was performed directly on the resin‐bound unsaturated peptide upon treatment with thioacetic acid and a suitable initiator/photosensitizer combination under UV irradiation at 365 nm. The reaction was initiated by the initiator/photosensitizer combination of 2,2‐dimethoxy‐2‐phenylacetophenone (DPAP) and 4‐methoxyacetophenone (MAP) in dimethylformamide (DMF), furnishing the corresponding resin‐bound peptide thioacetate **2**. Following standard resin washing and acidic peptide cleavage steps, the desired thioacetate **2** was isolated along with residual alkene starting material **1** (Table 1). Conversion to the thioacetate **2** was determined by ¹H NMR. Notably, no other side products were detected, highlighting the selectivity of the process. At shorter reaction times (5 min, Table 1, **entries 1** and **2**), conversion to the thioacetate **2** was moderate, even when higher equivalents of thioacetic acid and DPAP/MAP were employed. However, upon treatment with more forcing conditions, employing 20 equivalents of thioacetic acid along with 10 equivalents each of DPAP and MAP, >95% conversion to **2** was achieved within 15 min (Table 1, **entry 3**). Under these optimized conditions, the ¹H NMR (Figure 1B) indicated complete disappearance of the characteristic alkene signal (5.90–4.90 ppm) present in the starting material **1** (Figure 1A).

**Table 1:** Optimization table for the on‐resin ATE reaction between the unsaturated peptide **1** and thioacetic acid to yield peptide thioacetates **2** (x = NH_2_ at the *C*‐terminus using Rink Amide resin) and **3** (x = OH at the *C*‐terminus using Wang and 2‐ClTrt resins).

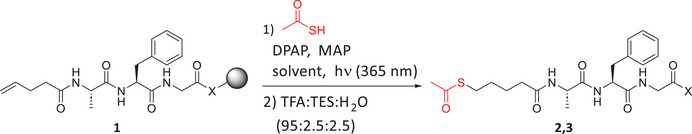						
Entry	Thioacetic acid	DPAP/MAP	Time	Solvent	Resin	Conversion
1	10 eq	5 eq	5 min	DMF	Rink Amide	44%
2	50 eq	25 eq	5 min	DMF	Rink Amide	52%
3	20 eq	10 eq	15 min	DMF	Rink Amide	>95%
4	20 eq	10 eq	15 min	NMP	Rink Amide	88%
5	20 eq	10 eq	15 min	CH_2_Cl_2_	Rink Amide	>95%
6	20 eq	10 eq	15 min	DMF	Wang	>95%
7	20 eq	10 eq	15 min	DMF	2‐ClTrt	>95%
8	20 eq	10 eq	25 min	DMF	Rink Amide	>95%

Using alternative solvents, including N‐methyl‐2‐pyrrolidone (NMP) and dichloromethane (CH₂Cl₂), both commonly used in SPPS, had minimal impact on reaction efficiency (Table 1, **entries 4** and **5**). Additionally, peptides synthesized on Wang and 2‐chlorotrityl (2‐ClTrt) resins, which enable isolation of *C*‐terminal peptide carboxylic acid **3**, were also compatible with these conditions (Table 1, **entries 6** and **7**). This is particularly significant for the 2‐ClTrt resin, as it allows for the isolation of fully protected peptides post‐SPPS, demonstrating the feasibility of ATE/*S*‐deacetylation strategy for the synthesis of fully protected, thiolated peptides and enabling further thiol modifications in solution.

Finally, the ATE reaction was initiated using a low‐power (36 W) nail lamp instead of a standard laboratory‐scale Luzchem photoreactor (Table 1, **entries 1–7**). Under optimized conditions (Table 1, **entry 8**), the nail lamp achieved full conversion within 25 min, demonstrating that on‐resin ATE methodologies and thiol‐ene reactions, generally, can be performed in laboratories without requiring specialized photochemical equipment.

Building on the optimized conditions established for the on‐resin ATE reaction, we next developed efficient conditions for on‐resin *S*‐deacetylation (Table 2). The resin‐bound thioacetate **2**, prepared through standard SPPS followed by the on‐resin ATE reaction, was exposed to various thiol‐containing reagents to promote *S*‐deacetylation. DMF was chosen as the solvent of choice due to its excellent resin‐swelling properties. Previous studies by Wan and co‐workers provided a basis for investigating *S*‐deacetylation conditions for anomeric thioacetates in thioglycoside synthesis.^[^ ^29^ ^]^ Initially, L‐cysteine hydrochloride methyl ester (HCl·H‐Cys‐OMe) was used to achieve irreversible *S*‐deacetylation of **2** via intermolecular *S*‐*S* acyl transfer. Triethylamine (TEA) was added as a base to neutralize cysteine hydrochloride salts and to facilitate proton transfer in the reaction medium. However, poor solubility of these additives in DMF resulted in only 20% conversion to the free thiol **4** (Table 2, **entry 1**). Next, dithiothreitol (DTT) was evaluated as an alternative thiol nucleophile due to its superior solubility in DMF. Treating resin‐bound thioacetate **2** with high equivalents of DTT and TEA improved the conversion to thiol **4**, reaching 56% (Table 2, **entry 2**). Further screening bases, including N‐methyl‐morpholine (NMM) and N,N’‐diisopropylethylamine (DIPEA), did not enhance the reaction yield (Table 2, **entries 3** and **4**). Extending the reaction duration increased the conversion to 78%, but a 3 h reaction time was impractical for SPPS, necessitating further optimization. Ultimately, a combination of DTT and HCl·H‐Cys‐OMe in a DMF:phosphate buffer (8:2) at pH 8.5 was identified as optimum, which not only supported efficient resin swelling but also improved reagent solubility and facilitated thiolate formation (Table 2, **entry 6**). Notably, treating resin‐bound peptide **2** with 100 equivalents of both DTT and HCl·H‐Cys‐OMe, respectively, in DMF:phosphate buffer (8:2) at pH 8.5 resulted in >95% conversion to the free thiol **4** within 1 h. Under these conditions, ^1^H NMR analysis of the crude mixture confirmed the disappearance of the characteristic singlet peak at 2.32 ppm (CH_3_CO‐) corresponding to the starting thioacetate **2**, furnishing the desired thiol **4** (Figure 1C).

**Table 2:** Optimization table for the on‐resin *S*‐deacetylation of peptide thioacetate **2** to yield thiolated peptide **4**.

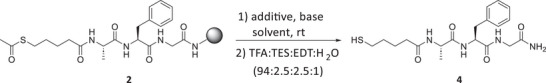					
Entry	Additive	Base	Time	Solvent	Conversion
1	HCl·H‐Cys‐OMe (10 eq)	TEA (10 eq)	60 min	DMF	20%
2	DTT (100 eq)	TEA (100 eq)	60 min	DMF	56%
3	DTT (100 eq)	DIPEA (100 eq)	60 min	DMF	40%
4	DTT (100 eq)	NMM (100 eq)	60 min	DMF	53%
5	DTT (100 eq)	TEA (100 eq)	180 min	DMF	78%
6	DTT (100 eq) + HCl·H‐Cys‐OMe (100 eq)	‐	60 min	DMF + PB (pH 8.5)	>95%

### 2.2 Development of New VP and SST Analogs

**Scheme 2:**
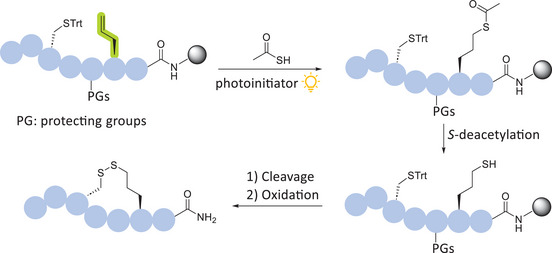
Synthetic workflow for the development of disulfide‐based neuropeptide analogs with increased ring sizes.

With the optimized on‐resin hydrothiolation/S‐deacetylation conditions in hand, we set out to investigate the application of the methodology toward developing novel neuropeptide analogs of VP and SST, maintaining the critical disulfide bonds while expanding the ring size. Through judicious substitution of either one or two cysteine residues in the native sequences with commercially available Allyl‐Gly amino acid, followed by a sequential ATE reaction with thioacetic acid and *S*‐deacetylation, the free thiol could be regioselectively installed into the peptide while on‐resin, offering improved ease of purification over other late‐stage modification methods (Scheme 2). Following acidic cleavage and global deprotection, the linear unprotected peptide was cyclized under oxidative conditions to furnish the desired disulfide‐based neuropeptide analog with an enlarged ring size (Scheme 2). This approach, utilizing the inexpensive and chemically stable Allyl‐Gly residue, offers a simple and reliable strategy for expanding the ring size of any disulfide‐containing bioactive cyclic peptide.

**Figure 2:**
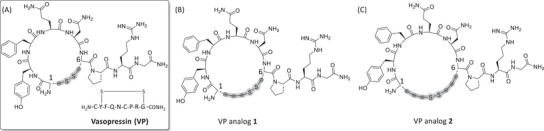
Chemical structures of A) VP, B) VP analog 1 bearing two additional carbon atoms (synthesized in this work), and C) VP analog 2 bearing four additional carbon atoms (synthesized in this work). Note: the chemical structures are presented with *cis* amide bonds for illustration purposes only and do not resemble the actual amide bond configuration, which is *trans*.

VP, also known as arginine vasopressin (AVP) or antidiuretic hormone (ADH), is a cyclic peptide hormone consisting of 9 amino acids, produced in the hypothalamus.^[^ ^30^, ^31^ ^]^ The cyclic structure of VP features a disulfide bond between cysteine residues at positions 1 (*N*‐terminus) and 6 (Figure 2A). The primary physiological functions of VP include promoting vasoconstriction in arterioles, which increases peripheral vascular resistance and consequently raises arterial blood pressure.^[^ ^32^ ^]^ We applied our developed method for direct on‐resin peptide hydrothiolation/*S*‐deacetylation to create two disulfide‐based VP analogs, **1** and **2**, with ring sizes extended by two and four carbons, respectively (Figures 2B and 2C).

**Scheme 3:**
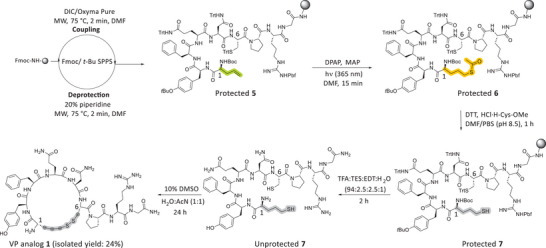
Synthetic route to produce VP analog 1.

VP analog **1** was designed to include two additional carbon atoms relative to the native structure, and the linear peptide was synthesized on a CEM Liberty Blue peptide synthesizer by replacing the cysteine residue at position 1 (*N*‐terminus) with Allyl‐Gly. The on‐resin ATE reaction was conducted using the optimized conditions (Table 1, **entry 3**), resulting in the regioselective introduction of the thioacetate group at the *N*‐terminus of the protected peptide **6** on‐resin (Scheme 3). The subsequent on‐resin *S*‐deacetylation was performed using the previously optimized conditions (Table 2, **entry 6**), furnishing a free thiol at the *N*‐terminus of the protected peptide **7** (Scheme 3). Following acidic cleavage and global deprotection, the linear unprotected peptide **7** was cyclized without further purification via disulfide bond formation. Cyclization via disulfide bond formation was performed using previously reported conditions (**SI**).^[^ ^33^, ^34^, ^35^ ^]^ Following semi‐preparative RP‐HPLC purification, the desired VP analog **1** was obtained in a 24% overall yield with >95% purity, as confirmed by analytical RP‐HPLC (Figures S10 and S11).

**Figure 3:**
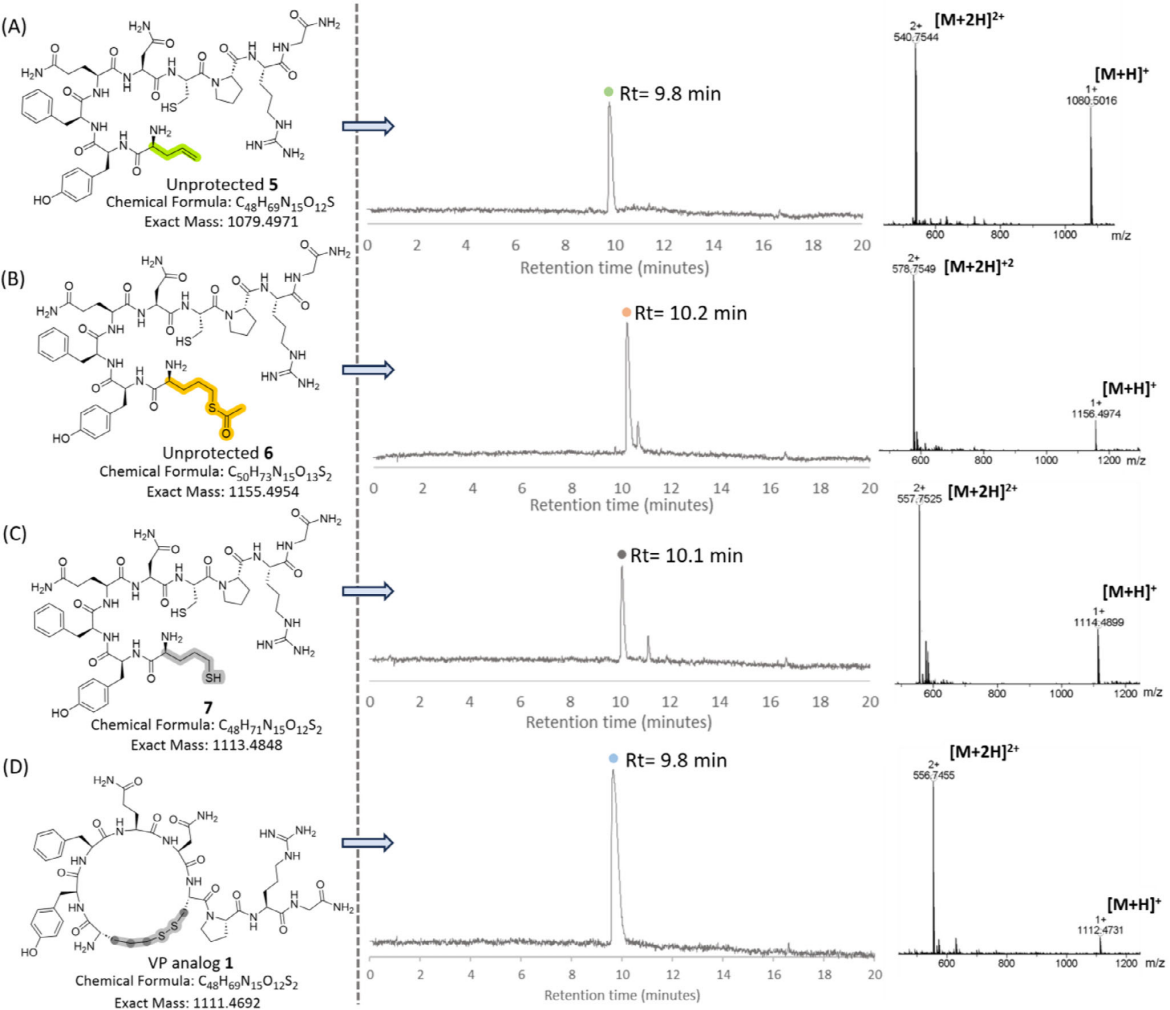
A) Analytical RP‐HPLC (220 nm) trace of the unprotected starting material 5. ESI‐MS analysis of the main peak with a retention time of 9.8 min confirmed the mass of the unprotected peptide 5 (calculated for C_48_H_70_N_15_O_12_S (M+H)^+1^ = 1080.5049; observed 1080.5016, calculated for C_48_H_71_N_15_O_12_S (M+2H)^+2^ = 540.7563; observed 540.7544). B) Analytical RP‐HPLC (220 nm) trace of the unprotected peptide 6. ESI‐MS analysis of the main peak with a retention time of 10.2 min confirmed the mass of the unprotected peptide 6 (calculated for C_50_H_74_N_15_O_13_S_2_ (M+H)^+1^ = 1156.4954; observed 1156.4974, calculated for C_50_H_75_N_15_O_13_S_2_ (M+2H)^+2^ = 578.7555; observed 578.7549). C) Analytical RP‐HPLC (220 nm) trace of the peptide 7. ESI‐MS analysis of the main peak with a retention time of 10.1 min confirmed the mass of the peptide 7 (calculated for C_48_H_72_N_15_O_12_S_2_ (M+H)^+1^ = 1114.4826; observed 1114.4899, calculated for C_48_H_73_N_15_O_12_S_2_ (M+2H)^+2^ = 557.7502; observed 557.7525). D) Analytical RP‐HPLC (220 nm) trace of VP analog 1. ESI‐MS analysis of the peak with a retention time of 9.8 min confirmed the mass of VP analog 1 (calculated for C_48_H_70_N_15_O_12_S_2_ (M+H)^+1^ = 1112.4770; observed 1112.4731, calculated for C_48_H_71_N_15_O_12_S_2_ (M+2H)^+2^ = 556.7424; observed 556.7455).

To track the progress of the two on‐resin reactions, small‐scale peptide cleavage and global deprotection were performed on each intermediate. The resulting linear unprotected intermediate products were analyzed by analytical RP‐HPLC and ESI‐MS. The linear unprotected starting material **5**, featuring a terminal alkene at the *N*‐terminus, was obtained with high purity (Figure 3A). The mass of the linear unprotected peptide **5** was identified under the main RP‐HPLC peak (Figure 3A). Following the first on‐resin ATE reaction and small‐scale cleavage and deprotection, the RP‐HPLC trace of the crude material revealed the complete consumption of the starting peptide **5** and the appearance of a new main peak, corresponding to the mass of the desired linear unprotected peptide **6** featuring the thioacetate group at the *N*‐terminus (Figure 3B). Following this, on‐resin *S*‐deacetylation was carried out, and the product was analyzed in the same manner. The RP‐HPLC trace of the crude material confirmed complete consumption of the starting unprotected thioacetate **6**, with a new main peak corresponding to the mass of the desired dithiol linear peptide **7** (Figure 3C). Notably, the analysis of the intermediate products confirmed the high efficiency of the two sequential on‐resin reactions for direct peptide thiolation. Both reactions proceeded quantitatively, yielding intermediate and final products in high purity (>90%).

**Scheme 4:**
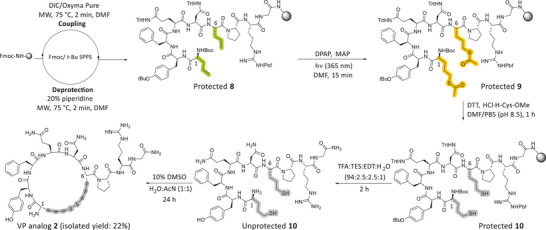
Synthetic route to produce VP analog 2.

VP analog **2** contains four additional carbon atoms relative to the native peptide. This modification was achieved by replacing both cysteine residues at positions 1 (*N*‐terminus) and 6 with Allyl‐Gly. The regioselective installation of thiol groups at both alkene sites was achieved using our optimized protocol, which, following peptide cleavage and deprotection, allowed for cyclization via disulfide bond formation (Scheme 4). VP analog **2** was isolated in a 22% overall yield with >95% purity, as confirmed by analytical RP‐HPLC (Figures S13 and S14). As with the synthesis of VP analog **1**, the progress of both on‐resin reactions was monitored through small‐scale peptide cleavage and deprotection, followed by an analysis of the intermediate unprotected products using analytical RP‐HPLC and ESI‐MS (Figure S1). Fortunately, both on‐resin reactions proceeded quantitatively, producing highly pure intermediate and final products. This demonstrated the scope and wide applicability of the developed methodology, which enables the direct on‐resin hydrothiolation of multiple sites within peptide sequences.

**Figure 4:**
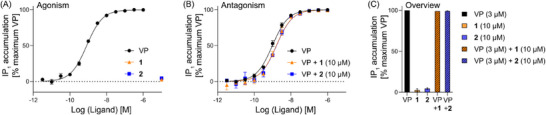
Biological evaluation of VP analogues 1 and 2. Functional IP_1_ assays were performed on stable HEK‐293 cells overexpressing the human V_1a_R. A) Single point measurement of 1/2 at 10 µM to determine potential agonism. B) Addition of 10 µM 1/2 to a dilution series of VP (3 µM–30 pM) to determine potential antagonism. C) Overview of IP_1_ assay results for agonism and antagonism. Each point represents three (agonism) or two (antagonism) independent measurements with technical triplicates. Results normalized to the highest VP activity (100%) and vehicle control (0%). Error bars indicate the standard error of the mean (SEM).

After successfully synthesizing the two VP analogs 1 and 2, we next investigated the impact of the ring size expansion on the activity of VP. Although the macrocyclic ring of VP interacts with residues deep inside the receptor binding pocket, some modifications are still tolerated.^[^ ^36^, ^37^, ^38^ ^]^ Subtle changes to the amino acid side chains, as well as, for example, the exchange of the disulfide bridge to diselenides, have led to potent ligands, whereas reduction of the ring size results in a complete loss of activity.^[^ ^39^, ^40^, ^41^, ^42^, ^43^ ^]^ To assess the derivatives’ ability to interact with this receptor, we carried out functional cellular assays (HTRF IP‐One G_q_ Detection Kit, Revvity) on HEK‐293 cells, stably overexpressing the human V_1a_ receptor (hV_1a_R). To check for agonism, we tested analogs 1 and 2 at 10 µM, but even at these high concentrations, no receptor activity above the baseline level was detected (Figure 4A/C). To further investigate if the compounds could possibly act as antagonists, we added 10 µM of 1 and 2 to the VP dilution series, but no competitive ligand displacement (shift of the sigmoidal curve to the right) was observed, indicating that the derivatives did not interact with the orthosteric binding pocket of the receptor (Figure 4B/C). These results further highlight the importance and sensitivity of the macrocyclic ring system, not only to reduction but also to increased ring size.

**Figure 5:**
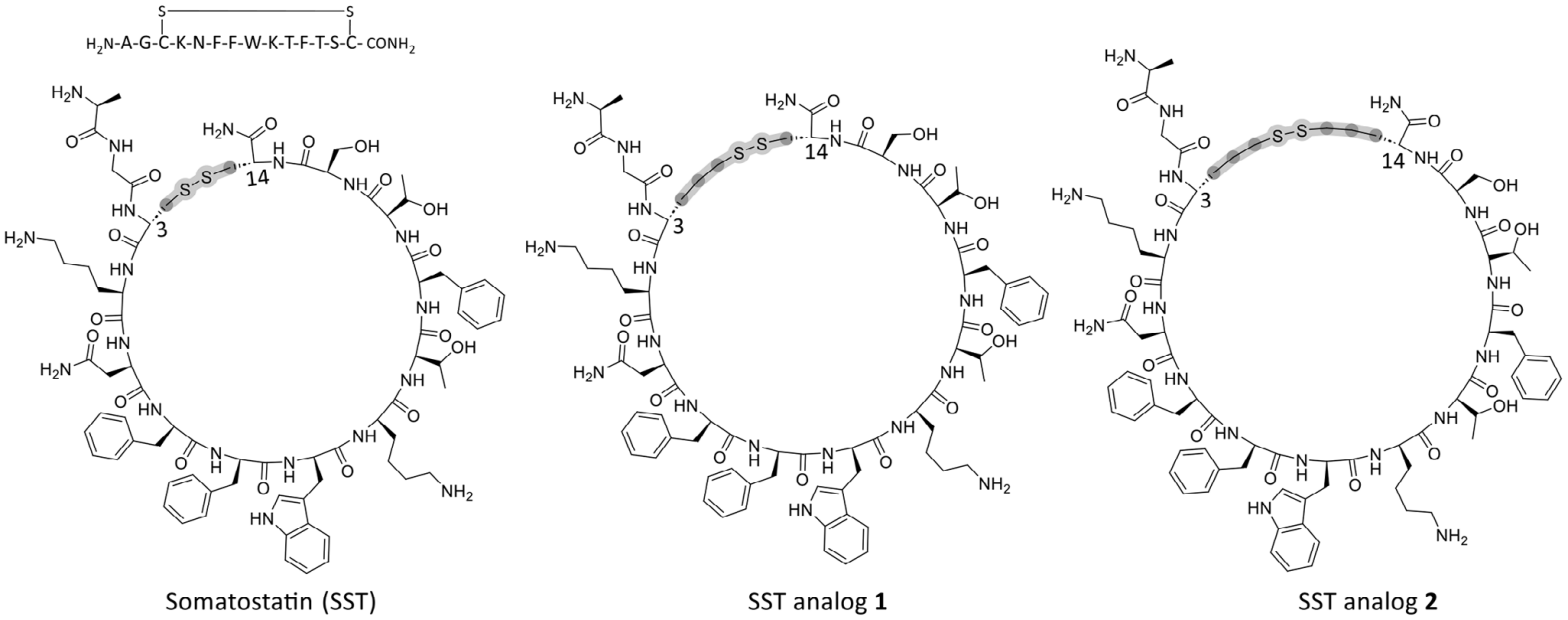
Chemical structures of SST and SST analogs 1 and 2, synthesized in this work. Note: the chemical structures are presented with *cis* amide bonds for illustration purposes only and do not resemble the actual amide bond configuration, which is *trans*.

SST, also referred to as growth hormone‐inhibiting hormone, is a cyclic polypeptide produced in various regions of the human body, including the hypothalamus, pancreas, gastrointestinal tract, and central nervous system.^[^ ^44^ ^]^ Its structure consists of fourteen amino acids, with a disulfide bridge between the cysteine residues at positions 3 and 14 (Figure 5). SST exerts biological effects by binding to specific G protein‐coupled receptors (SSTR1‐5).^[^ ^45^ ^]^ These interactions inhibit the secretion of pancreatic, exocrine, and pituitary hormones, as well as suppress angiogenesis and the proliferation of cancer cells. Similar to the synthetic strategy employed for the VP analogs, two novel SST analogs with expanded ring sizes by two and four carbons were produced (Figure 5).

**Figure 6:**
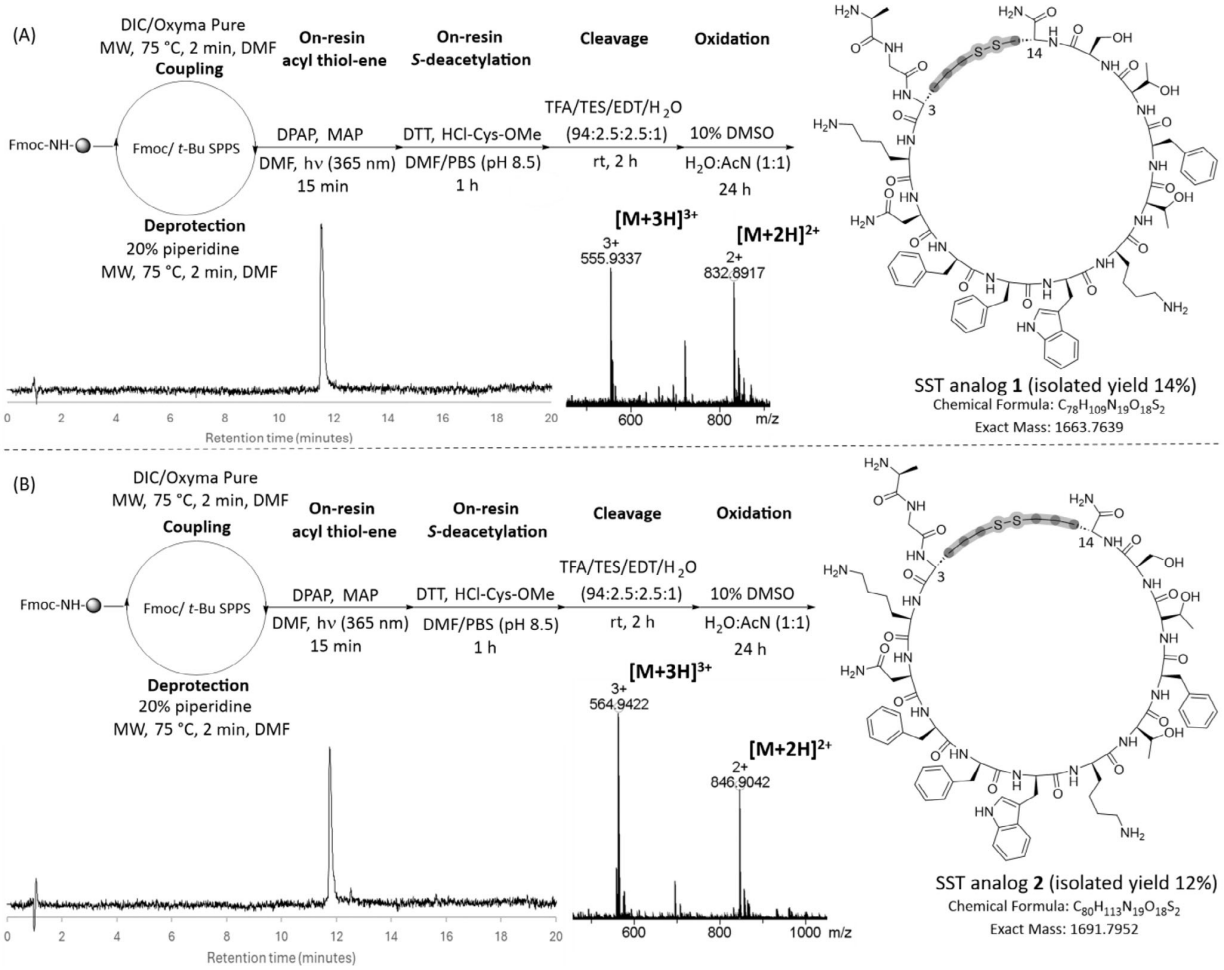
A) Synthetic route to produce SST analog **1**. Analytical RP‐HPLC (220 nm) trace and ESI‐MS analysis of the pure SST analog **1** (calculated for C_78_H_111_N_19_O_18_S_2_ (M+2H)^+2^ = 832.8859; observed 832.8896, calculated for C_78_H_112_N_19_O_18_S_2_ (M+3H)^+3^ = 555.9353; observed 555.9337). B) Synthetic route to produce SST analog **2**. Analytical RP‐HPLC (220 nm) trace and ESI‐MS analysis of the pure SST analog **2** (calculated for C_80_H_115_N_19_O_18_S_2_ (M+2H)^+2^ = 846.9054; observed 846.9042, calculated for C_80_H_116_N_19_O_18_S_2_ (M+3H)^+3^ = 564.9395; observed 564.9422).

SST analogs 1 and 2 were accessed by systematically replacing cysteine residues with Allyl‐Gly, followed by direct peptide hydrothiolation, and final cyclization via disulfide bond formation in solution. For SST analog 1, only the cysteine residue at position 3 was substituted with Allyl‐Gly, while in SST analog 2, both cysteine residues were replaced with Allyl‐Gly. Ultimately, both SST analogs 1 and 2 were isolated with high purity (>95%), as confirmed by analytical RP‐HPLC (Figure 6A,B). This demonstrated the broader applicability of the new methodology, which can be employed for larger and more complex peptide structures.

### 2.3 On‐Resin Bioconjugation

**Figure 7:**
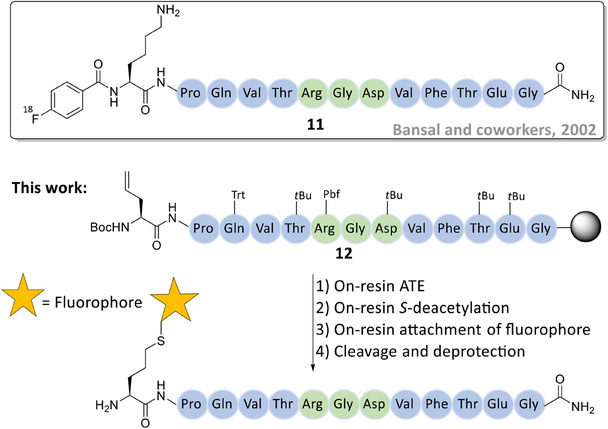
Application of the on‐resin hydrothiolation strategy for the covalent attachment of a fluorescence tag onto the linear RGD peptide 12 prior to peptide cleavage and deprotection.

To further broaden the application of the on‐resin hydrothiolation/*S*‐deacetylation protocol, our method was applied to fluorescent labeling of a linear peptide displaying the RGD motif. Peptides with the RGD sequence bind strongly to the αvβ3 integrin receptor, which is overexpressed in many cancer cells.^[^ ^46^ ^]^ Consequently, RGD‐based peptide agents are commonly used in cancer treatments for therapeutic and diagnostic purposes.^[^ ^28^ ^]^ Bansal and co‐workers reported an ^18^F‐labeled linear RGD peptide (KPQVTRGDVFTEG, **11**) to study its biodistribution in tumor‐bearing mice.^[^ ^47^ ^]^ The RGD sequence was centrally positioned within the peptide sequence (shown in green, Figure 7), with 4‐[^18^F] fluorobenzoic acid covalently attached to the *N*‐terminal lysine. This peptide tracer was considered a suitable candidate for on‐resin hydrothiolation/*S*‐deacetylation and the subsequent covalent attachment of a fluorescence label via the free thiol group. In this study, the *N*‐terminal lysine of the peptide was replaced with Allyl‐Gly and, following on‐resin hydrothiolation, the exposed thiol group at the *N*‐terminus became a reactive site for the covalent attachment of a fluorophore prior to peptide cleavage and deprotection.

**Scheme 5:**
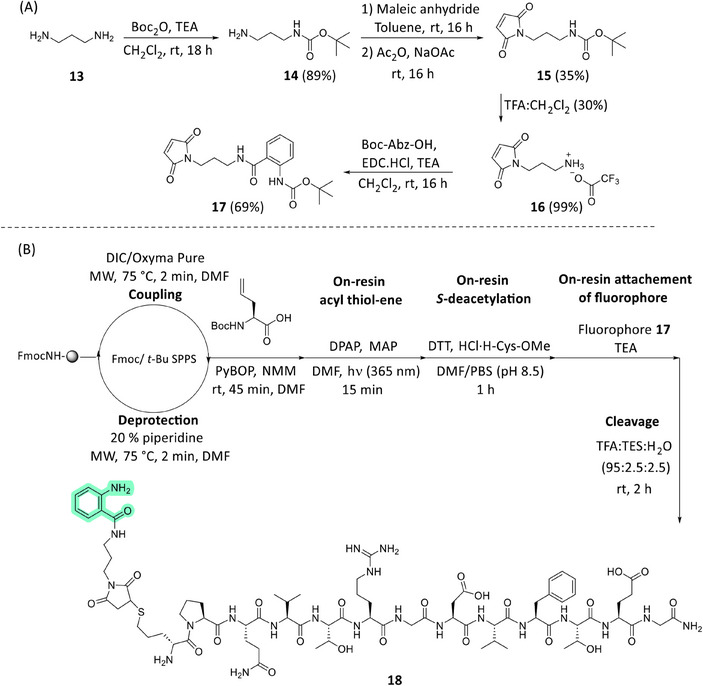
A) Synthetic route to produce the protected fluorophore 17. B) Synthetic route to produce peptide 18 bearing the Abz group at the *N*‐terminus.

Labeling the peptide with an ortho‐aminobenzoic acid (Abz), fluorophore was explored in the first instance. The Abz fluorophore was connected via a small diamino spacer to a maleimide group, which is highly reactive in the presence of free thiols. Numerous maleimide‐containing fluorophores are commercially available, highlighting the generality of this approach. The synthesis of the fluorophore **17** commenced with Boc protection of the diaminopropane **13**, furnishing the free amine **14**, which was subsequently condensed with maleic anhydride to form compound **15**. Boc deprotection of **15** furnished the free amine **16**, which was then coupled with the commercially available Boc‐Abz‐OH fluorescent molecule, ultimately yielding the fluorophore **17**, which was used for direct covalent attachment on‐resin with peptide **12** to furnish the RGD analog **18** (Scheme 5B). Gratifyingly, following peptide cleavage and deprotection, the analytical RP‐HPLC trace of the crude mixture revealed only one main peak, corresponding to the mass of the desired final peptide **18**, with no intermediate products detected (Figure S22). This method allowed the covalent attachment of the fluorophore via three sequential, quantitative on‐resin reactions without the need for intermediate purification, opening possibilities for chemoselective advances in peptide bioconjugation, beyond the current state‐of‐the‐art. Future research will aim to extend this strategy for on‐resin conjugation with sugars, lipids, and cytotoxic agents using the free thiol group of biologically relevant peptides.

## 3 Conclusion

In this work, we developed a novel synthetic strategy for direct peptide hydrothiolation employing two simple quantitative on‐resin reactions. This was achieved by applying sequential ATE and *S*‐deacetylation chemistries on‐resin to regioselectively install a thiol group onto unsaturated peptide precursors. Optimized conditions were applied to the high‐yielding synthesis of novel, ring‐expanded analogs of VP and SST, which would be challenging to access using traditional late‐stage synthetic approaches. Finally, the methodology was employed for broadly applicable and convenient peptide bioconjugation by utilizing the free thiol group as a chemical handle to attach moieties of interest prior to peptide cleavage and deprotection. An RGD‐containing peptide was used as a proof‐of‐concept to attach the Abz fluorophore following the on‐resin hydrothiolation. In summary, we report the application of the photochemical acyl thiol‐ene reaction, with sequential *S*‐deacetylation, for synthesizing thiolated peptides starting from linear peptides bearing Allyl‐Gly residues. The reaction is chemo‐ and regioselective and represents the first reported synthesis of ring‐expanded neuropeptides under photochemical, radical‐mediated conditions. The rapid nature of the acyl thiol‐ene/S‐deacetylation protocol is expected to be highly useful for the generation of peptide libraries, particularly for the development of disulfide or thioether‐containing cyclic peptides. Further studies toward applying this methodology to prepare libraries of novel neuropeptide analogs suitable for high‐throughput screening are currently ongoing in our labs.

## Supporting Information

The authors have cited additional references within the Supporting Information.^[^ ^48^, ^49^, ^50^, ^51^ ^]^

## Conflict of Interests

The authors declare no conflict of interest.

## Supporting information



Supporting Information

## Data Availability

The data that support the findings of this study are available in the supplementary material of this article.
