# Supplementary material for: An Efficient Thiol‐ene Mediated Protocol for Thiolated Peptide Synthesis and On‐Resin Diversification
Source: Chemistry. 2025 May 23;31(36):e202501372. doi: 10.1002/chem.202501372 (PMC12202855; doi:10.1002/chem.202501372)
Supplement: Supplementary file 1 — Supporting Information [file CHEM-31-e202501372-s001.docx]

Table of Contents

[General Experimental Details S3](#_Toc194861118)

[General Experimental Procedures S4](#_Toc194861119)

[Peptide synthesis S4](#_Toc194861120)

[Cell assay S6](#_Toc194861121)

[Characterization Data S7](#_Toc194861122)

[NMR Spectra and Analytical PR-HPLC Traces. S18](#_Toc194861123)

[References S27](#_Toc194861124)

# General Experimental Details

All commercial chemicals used were supplied by Thermo Fisher Scientific, Sigma Aldrich (Merck), Fluorochem, and Tokyo Chemical Industry and used without further purification unless otherwise stated. Solvents for synthesis and purification were supplied by Sigma-Aldrich at HPLC grade. All UV reactions were carried out in a Luzchem photoreactor, LZC-EDU (110 V/ 60 Hz) containing 10 UVA lamps centred at 365 nm. Analytical thin layer chromatography (TLC) was carried out with silica gel 60 (fluorescence indicator F254; Merck) and visualized by ninhydrin staining or molybdenum staining. Silica gel 60 (Merck, 230-400 mesh) was used for silica gel flash chromatography and all compounds were subject to purification using silica gel, unless otherwise stated. NMR spectra were recorded using Bruker AV 600 (600.13 MHz for ^1^H NMR and 150.90 MHz for ^13^C NMR). Deuterated DMSO for NMR was purchased from Sigma Aldrich (Merck). Chemical shifts, δ, are in ppm and referenced to the internal solvent signals (DMSO at 2.50 ppm). ESI mass spectra were acquired using a Bruker micrOTOF-Q III spectrometer interfaced to a Dionex UltiMate 3000 LC in positive and negative modes as required. Agilent ESI-L Low Concentration Tuning Mix was used to calibrate the system, and this was also used as an internal lock mass. Operating conditions were as follows: end-plate offset 500 V, capillary 4500 V, nebulizer 2.0 bar, dry gas 8.0 L min−1, and dry temperature 180 °C. APCI measurements were conducted on a Bruker micrOTOF-Q III spectrometer interfaced with a Dionex UltiMate 3000 LC or a direct insertion probe, operating in either positive or negative modes.RP-HPLC analysis was performed using a Shimadzu Nexera Lite Low Pressure Gradient system. For analytical HPLC an Ascentis® Express 90 Å C18 column (100 mm × 4.6 mm, 5 μm) with a flow rate of 1.0 mL/min was utilized. For semi-preparative RP-HPLC purification, an Ascentis® C18 column (250 mm × 10 mm, 5 μm) with a flow rate of 4 mL/min was used. Gradient elution was carried out using Solvent A (0.1% TFA in water) and Solvent B (0.1% TFA in acetonitrile) for both analytical and semi-preparative HPLC runs. UV absorption signals were detected using a SPD-M40 PDA detector at suitable wavelengths (220 nm and 254 nm).

# General Experimental Procedures

## Peptide synthesis

***Procedure A: Manual SPPS of Linear Peptides***

Rink amide resin (loading: 0.68 mmol, 100 - 200 mesh) was added to the SPPS syringe, swollen in DMF (5 mL) for 30 min, and then drained. The resin was washed with DMF (3 × 5 mL), CH_2_Cl_2_ (3 × 5 mL), and DMF (3 × 5 mL). Fmoc deprotection was carried out with a solution of 20% piperidine (v/v) in DMF (5 mL) for 2 × 10 min and the resin was washed with DMF (3 × 5 mL), CH_2_Cl_2_ (3 × 5 mL), and DMF (3 × 5 mL). The first amino acid was coupled by preparing a solution of PyBOP (4 equiv.) and NMM (8 equiv.) and Fmoc protected amino acid (4 equiv.; 0.2 M) in DMF, and then adding it to the resin for 1 h. Subsequently, the solution was drained, and the resin as washed with DMF (3 × 5 mL), CH_2_Cl_2_ (3 × 5 mL), and DMF (3 × 5 mL). The subsequent amino acid coupling cycle is consisted of 1) Fmoc deprotection by adding a solution of 20% (v/v) piperidine in DMF (5 mL) to the resin for 2 x 10 min, 2) resin washes with DMF (3 × 5 mL), CH_2_Cl_2_ (3 × 5 mL), and DMF (3 × 5 mL), 3) amino acid coupling by adding a solution of PyBOP (4 equiv.), NMM (8 equiv.), and Fmoc protected AA (4 equiv.; 0.2 M) in DMF to the resin for 1 h, 4) resin washes with DMF (3 × 5 mL), CH_2_Cl_2_ (3 × 5 mL), and DMF (3 × 5 mL).

***Procedure B: Automated SPPS of Linear Peptides***

All linear peptide sequences were assembled by automated SPPS on a CEM Liberty Blue™ Automated Microwave Peptide Synthesiser (CEM Corporation, Buckingham, UK) using repetitive steps of Fmoc deprotections using a solution of 20% piperidine in DMF (at 75 °C for 1 min) and amino acid couplings with a solution of Fmoc-protected amino acid (0.2 M in DMF), DIC (0.5 M in DMF) and Oxyma Pure (1 M in DMF) at 75 °C for 2 min, with intermediate washes with DMF (3 × 30 s).

***Procedure C: On-resin ATE Reaction (optimized conditions)***

DPAP (0.128 g, 0.5 mmol, 10.0 equiv.), MAP (0.075 g, 0.5 mmol, 10.0 equiv.), and thioacetic acid (71 μL, 1 mmol, 20.0 equiv.) were added to a suspension of the resin bound unsaturated peptide (1.0 equiv. of peptide, 0.05 mmol) in DMF (5 mL) in a transparent glass vial. After complete solubilization of reagents, a LBX Orb-B2 shaker was used inside the photoreactor to ensure continuous agitation, and the suspended resin-bound peptide was irradiated at 365 nm for 15 min. Following this, the suspension was transferred to a SPPS syringe, and the solvent and excess of reagents were removed *via* filtration and thorough washings with DMF (3 × 5 mL), CH_2_Cl_2_ (3 × 5 mL), and DMF (3 × 5 mL). The resin was finally washed with CH_2_Cl_2_ and dried under vacuum. The progress of the reaction was checked by conducting small-scale peptide cleavage and deprotection by treating a small amount of the resin bound peptide with 1 mL of TFA:TES:EDT:H_2_O (94:2.5:2.5:1 v/v/v/v) for 2 h. The solvent was removed under reduced pressure and the crude peptide material was analyzed by analytical RP-HPLC.

***Procedure D: On-resin S-deacetylation reaction (optimized conditions)***

DTT (1.54 g, 10 mmol, 100.0 equiv.) and HCl·H-Cys-OMe (1.71 g, 10 mmol, 100.0 equiv.) were added to a suspension of the resin bound peptide (1.0 equiv. of peptide, 0.1 mmol) in 3 mL of DMF/Phosphate buffer (pH 8.5) (8:2 v/v) and the system was agitated for 1 h. The solvent and excess of reagents were removed *via* filtration and the resin was washed thoroughly with DMF (3 × 5 mL), CH_2_Cl_2_ (3 × 5 mL), and DMF (3 × 5 mL). The resin was finally washed with CH_2_Cl_2_ and dried under vacuum. The progress of the reaction was monitored as described in Procedure C.

***Procedure E: Resin cleavage and global deprotection***

CH_2_Cl_2_ (5 mL) was added to the resin-bound peptide, the syringe was agitated for 20 min, and then drained. A freshly prepared cleavage cocktail (TFA:TES:EDT:H_2_O; 94:2.5:2.5:1 v/v/v/v; 10 mL) was added to the resin and it was agitated for 2 h. The syringe was drained, and the filtrate was collected. The resin was washed with fresh cleavage cocktail (2 x 3 mL) and the washings were combined. The combined filtrate solution was concentrated under steam of N_2_, followed by precipitation of the peptide with cold Et_2_O (10 mL) at 0 °C. The crude peptide suspension was centrifuged, and the supernatant was decanted. The peptide was washed again with cold Et_2_O (3 x 10 mL), centrifuged, and collected. The crude peptide was lyophilized.

***Procedure F: Peptide Cyclization via Disulfide Formation***

The linear peptide precursor was dissolved in a solution of H_2_O/AcN (1:1, 1 mM) with 10% DMSO used as an oxidizing agent. The reaction mixture was stirred at rt for 24 h and the crude material was subjected directly to purification by semi-preparative RP-HPLC.

## Cell assay

All reagents were obtained from Thermo Fisher Scientific, Sigma-Aldrich, or Merck. Human embryonic kidney cells (HEK-293) expressing a green fluorescent protein (GFP)-tagged variant of the hV_1a_R G_q_-GPCR under the control of the same promoter as a Neomycin-Geneticin resistance gene were cryopreserved in 1 mL of 5% dimethyl sulfoxide (DMSO) in fetal bovine serum (FBS) and stored in liquid nitrogen. Upon retrieval, the cells were thawed and diluted 1:9 in Dulbecco’s Modified Eagle Medium (DMEM) supplemented with 10% FBS, 2 mM L-glutamine, and 1 mM sodium pyruvate. The cell suspension was centrifuged (3 min at 164 *g*), and the supernatant was aspirated. The cell pellet was resuspended in growth medium, seeded into T-25 cell culture flasks, and incubated at 37°C with 5% CO_2_ for 24 hours.

Subsequently, the medium was replaced with a selection medium containing DMEM, 10% FBS, 2 mM L-glutamine, 1 mM sodium pyruvate, and 160 µL/10 mL G-418 sulfate antibiotic (Geneticin, 50 mg/mL). Cells were incubated until they reached 80–90% confluency, then washed with phosphate-buffered saline (PBS), detached using 1.5 mL StableCell trypsin solution (0.5 g porcine trypsin and 0.2 g ethylenediaminetetraacetic acid (EDTA), 4 Na/L in Hanks' Balanced Salt Solution with phenol red), and centrifuged (3 min at 164 *g*). The supernatant was removed, the pellet was resuspended in the selection medium, and the cells were seeded into T-75 cell culture flasks.

The IP_1_-accumulation assay (HTRF IP-One Gq kit, Revvity) is a Förster resonance energy transfer (FRET)-based, time-resolved fluorescence assay that measures endogenous IP_1_ formation upon receptor activation. Lithium chloride (LiCl) was added to inhibit IP1 degradation to D-myo-inositol, facilitating IP1 accumulation. Excitation at 340 nm of a FRET donor (anti-IP_1_-Eu-Cryptate) resulted in fluorescence at 620 nm. If a FRET acceptor (d2-IP_1_) was bound to the antibody’s binding site, a bathochromically shifted FRET signal at 665 nm was detected. Endogenous IP_1_ competed with d2-IP_1_, leading to attenuation of the FRET signal, which was used to quantify IP_1_ levels and, thus, receptor activation.^48^

Stock solutions (1 mM) of VP analogs **1** and **2** were prepared in 30% ACN/ddH_2_O, while VP was prepared in ddH_2_O. These stocks were stored at -20 °C. Prior to the assay, stocks were thawed, and semi-logarithmically spaced VP dilutions (3 µM–30 pM) were prepared in the provided stimulation buffer. Agonism experiments used 10 µM **1** and **2** in stimulation buffer, whereas antagonism experiments included 10 µM **1** or **2** in all wells of the VP dilution series.

The assay followed the manufacturer’s protocol. Agonism experiments were performed in biological triplicates and antagonism experiments in biological duplicates. Cells were detached at 80–90% confluency, centrifuged, and the supernatant aspirated. The pellet was resuspended in the selection medium, and cells were counted using a Neubauer counting chamber. 10,000 cells per well in 50 µL selection medium were seeded into sterile, white polystyrene 384-well plates (Greiner, tissue culture-treated, flat-bottom) and incubated for 48 hours. On the assay day, the medium was removed by pressing the plate onto tissue paper, and 5 µL of stimulation buffer was added per well. After a 15-minute incubation, 5 µL of the test compounds were added. VP served as a positive control, and stimulation buffer as a negative control. Each concentration was tested in technical triplicates.

After a 1-hour incubation, 5 µL of d2-IP_1_ and anti-IP_1_-cryptate in lysis and detection buffer were sequentially added. The plate was equilibrated at room temperature for 1 hour before readout on a Tecan Spark multimode microplate reader (Tecan, Männedorf, Switzerland). Emission ratios (665 nm/620 nm) after 340 nm excitation were recorded, analyzed using GraphPad Prism 9, and normalized to the highest positive control concentration (100% = 3 µM VP) and the negative control (0% = stimulation buffer).

# Characterization Data

***S*-(5-(((*S*)-1-(((*S*)-1-((2-amino-2-oxoethyl)amino)-1-oxo-3-phenylpropan-2-yl)amino)-1-oxopropan-2-yl)amino)-5-oxopentyl) ethanethioate (1)**

The peptide was synthesized at 0.10 mmol scale by manual SPPS as described in Procedure A using Fmoc-Gly-OH, Fmoc-Phe-OH, Fmoc-Ala-OH, and 4-pentenoic acid. Resin cleavage and global deprotection of the peptide were carried out as described in Procedure E, yielding the tetrapeptide product **1** (28 mg, 75%).

**^1^H NMR** (600 MHz, DMSO-d_6_): δ_H_ 8.11 (t, *J* = 5.7 Hz, 1H, Gly NH), 8.02 (d, *J* = 6.4 Hz, 1H, Ala NH), 7.99 (d, *J* = 7.7 Hz, 1H, Phe NH), 7.26-7.17 (m, 5H, Phe ArH), 7.10 (d, ­*J*­ = 4.1 Hz, 2H, C=ONH_2_), 5.82 – 5.76 (m, 1H, CH_2_=CH), 5.01 (d, *J* = 17.2 Hz, 1H, CH_2_=CH x1), 4.94 (d, *J* = 10.2 Hz, 1H, CH_2_=CH x1), 4.45 – 4.41 (m, 1H, Phe αCH), 4.21 – 4.16 (m, 1H, Ala αCH), 3.69 (dd, *J* = 16.6, 5.8 Hz, 1H, Gly αCH x1), 3.55 (dd, *J* = 16.6, 5.8 Hz, 1H, Gly αCH x1), 3.08 (dd, ­*J* = 14.0, 4.8 Hz, 1H, Phe βCH_2_ x1), 2.85 (dd, ­*J* = 14.0, 9.2 Hz, 1H, Phe βCH_2_ x1), 2.24 – 2.15 (m, 4H, CH_2_-CH_2_), 1.11 (d, *J* = 7.0 Hz, Ala CH_3_).

**^13^C NMR** (151 MHz, DMSO-d_6_): δ_C_ 172.5 (Ala C=O), 171.6 (Pent C=O), 171.0 (Phe C=O), 170.7 (Gly C=O), 137.8 (Phe qC), 137.7 (CH_2_=CH), 129.1 (Phe Ar C), 128.0 (Phe Ar C), 126.2 (Phe Ar C), 114.9 (CH_2_=CH), 54.0 (Phe αCH), 48.3 (Ala αCH), 42.0 (Gly αCH), 36.9 (Phe βCH_2_), 34.1 (C=O-CH_2_), 29.0 (C=O-CH_2_-CH_2_), 17.7 (Ala CH_3_).

**Analytical RP-HPLC R_t_** (5 – 95% AcN in H_2_O with 0.1% (v/v) TFA over 20 min, 220 nm and 254 nm): 8.96 min.

**HRMS** (m/z ESI^+^): calculated for C_19_H_27_N_4_O_4_ (M+H)^+1^= 375.2027; observed 375.2022).

***S*-(5-(((*S*)-1-(((*S*)-1-((2-amino-2-oxoethyl)amino)-1-oxo-3-phenylpropan-2-yl)amino)-1-oxopropan-2-yl)amino)-5-oxopentyl) ethanethioate (2)**

The peptide **1** was synthesized at 0.10 mmol scale by manual SPPS as described in Procedure A using Fmoc-Gly-OH, Fmoc-Phe-OH, Fmoc-Ala-OH, and 4-pentenoic acid. On-resin ATE reaction was carried out as described in Procedure C. Resin cleavage and global deprotection of the peptide were carried out as described in Procedure E, yielding the tetrapeptide product **2** (38 mg, 84%).

**^1^H NMR** (600 MHz, DMSO-d_6_): δ_H_ 8.11 (t, *J* = 5.8 Hz, 1H, Gly NH), 8.01 – 7.98 (m, 2H, Ala NH, Phe NH), 7.27 – 7.16 (m, 5H, Phe ArH), 7.11 – 7.08 (m, 2H, C=ONH_2_), 4.45 – 4.40 (m, 1H, Phe αCH), 4.18 (quin, ­*J* = 6.8 Hz, 1H, Ala αCH), 3.68 (dd, *J* = 16.8, 5.8 Hz, 1H, Gly αCH x1), 3.55 (dd, *J* = 16.8, 5.8 Hz, 1H, Gly αCH x1), 3.08 (dd, ­*J* = 13.7, 5.0 Hz, 1H, Phe βCH_2_ x1), 2.88 – 2.80 (m, 3H, Phe βCH_2_ x1, S-CH_2_), 2.32 (s, 3H, C=OCH_3_), 2.11 – 2.07 (m, 2H, C=OCH_2_), 1.54 – 1.42 (m, 4H, CH_2_-CH_2_), 1.11 (d, *J* = 7.0 Hz, Ala CH_3_).

**^13^C NMR** (151 MHz, DMSO-d_6_): δ_C_ 195.3 (SC=O),172.5 (Ala C=O), 172.0 (Pent C=O), 171.0 (Phe C=O), 170.7 (Gly C=O), 137.8 (Phe qC), 129.2 (Phe Ar C), 128.0 (Phe Ar C), 126.2 (Phe Ar C), 54.0 (Phe αCH), 48.3 (Ala αCH), 42.0 (Gly αCH), 36.9 (Phe βCH_2_), 34.4 (C=O-CH_2_), 30.6 (C=OCH_3_), 28.7 (S-CH_2_-CH_2_), 28.1 (S-CH_2_), 24.2 (C=OCH_2_), 17.7 (Ala CH_3_).

**Analytical RP-HPLC R_t_** (5 – 95% AcN in H_2_O with 0.1% (v/v) TFA over 20 min, 220 nm and 254 nm): 9.97 min.

**HRMS** (m/z ESI^+^): calculated for C_21_H_32_N_4_O_5_ (M+H)^+1^= 451.2010; observed 451.2015).

***N*-((*S*)-1-(((*S*)-1-((2-amino-2-oxoethyl)amino)-1-oxo-3-phenylpropan-2-yl)amino)-1-oxopropan-2-yl)-5-mercaptopentanamide (4)**

The peptide **1** was synthesized at 0.10 mmol scale by manual SPPS as described in Procedure A using Fmoc-Gly-OH, Fmoc-Phe-OH, Fmoc-Ala-OH, and 4-pentenoic acid. ATE and *S*-deacetylation reactions were carried out on-resin as described in Procedures C and D, respectively. Resin cleavage and global deprotection of the peptide were carried out as described in Procedure E, yielding the tetrapeptide product **4** (31 mg, 76%).

**^1^H NMR** (600 MHz, DMSO-d_6_): δ_H_ 8.13 – 8.10 (m, 1H, Gly NH), 8.02 – 7.96 (m, 2H, Ala NH, Phe NH), 7.26 – 7.17 (m, 5H, Phe ArH), 7.09 (d, *J* = 8.0 Hz, 2H, C=ONH_2_), 4.45 – 4.41 (m, 1H, Phe αCH), 4.21 – 4.18 (m, 1H, Ala αCH), 3.68 (dd, *J* = 16.8, 5.8 Hz, 1H, Gly αCH x1), 3.55 (dd, *J* = 16.8, 5.8 Hz, 1H, Gly αCH x1), 3.08 (dd, ­*J* = 13.9, 5.2 Hz, 1H, Phe βCH_2_ x1), 2.87 – 2.83 (m, 1H, Phe βCH_2_ x1), 2.46 (q, *J* = 6.9, 2H, SH-CH_2_) 2.22 (t, *J* = 7.5 Hz, 1H, SH), 2.14-2.07 (m, 2H, C=OCH_2_), 1.60-1.46 (m, 4H, CH_2_-CH_2_), 1.11 (d, *J* = 7.3 Hz, Ala CH_3_).

**^13^C NMR** (151 MHz, DMSO-d_6_): δ_C_ 172.5 (Ala C=O), 172.0 (Pent C=O), 171.0 (Phe C=O), 170.7 (Gly C=O), 137.8 (Phe qC), 129.1 (Phe Ar C), 128.0 (Phe Ar C), 126.2 (Phe Ar C), 54.0 (Phe αCH), 48.3 (Ala αCH), 41.9 (Gly αCH), 36.9 (Phe βCH_2_), 34.4 (C=O-CH_2_), 32.9 (SH-CH_2_-CH_2_), 23.8 (C=O-CH_2_-CH_2_), 23.5 (SH-CH_2_), 17.7 (Ala CH_3_).

**Analytical RP-HPLC R_t_** (5 – 95% AcN in H_2_O with 0.1% (v/v) TFA over 20 min, 220 nm and 254 nm): 9.36 min.

**HRMS** (m/z ESI^+^): calculated for C_19_H_28_N_4_O_4_SNa (M+Na)^+1^= 431.1723; observed 431.1718.

**VP Analog 1**

The peptide was synthesized at 0.10 mmol scale by automated SPPS as described in Procedure B using Fmoc-Gly-OH, Fmoc-Arg(Pbf)-OH, Fmoc-Pro-OH, Fmoc-Cys(Trt)-OH, Fmoc-Asn(Trt)-OH, Fmoc-Gln(Trt)-OH, Fmoc-Phe-OH, Fmoc-Tyr(*t*Bu)-OH, and Boc-AgI-OH. ATE and *S*-deacetylation reactions were carried out on-resin as described in Procedures C and D, respectively. Resin cleavage and global deprotection of the peptide were carried out as described in Procedure E. The linear peptide precursor was cyclized *via* disulfide bond formation as described in Procedure F. The reaction mixture was subjected directly to purification by semi-preparative RP-HPLC (5 – 50% AcN in H_2_O with 0.1% (v/v) TFA over 40 min.) yielding the desired final product as a white solid (26 mg, isolated yield: 24%, >95% purity).

**Analytical RP-HPLC R_t_** (5 – 95% AcN in H_2_O with 0.1% (v/v) TFA over 20 min, 220 nm and 254 nm): 9.827 min.

**HRMS** (*m/z* ESI^+^): calculated for C_48_H_70_N_15_O_12_S_2_ (M+H)^+1^= 1112.4770; observed 1112.4731, calculated for C_48_H_71_N_15_O_12_S_2_ (M+2H)^+2^ = 556.7424; observed 556.7455.

**VP Analog 2**

The peptide was synthesized at 0.1 mmol scale by automated SPPS as described in Procedure B using Fmoc-Gly-OH, Fmoc-Arg(Pbf)-OH, Fmoc-Pro-OH, Fmoc-AgI-OH, Fmoc-Asn(Trt)-OH, Fmoc-Gln(Trt)-OH, Fmoc-Phe-OH, Fmoc-Tyr(*t*Bu)-OH, and Boc-AgI-OH. ATE and *S*-deacetylation reactions were carried out on-resin as described in Procedures C and D, respectively. Resin cleavage and global deprotection of the peptide were carried out as described in Procedure E. The linear peptide precursor was cyclized *via* disulfide bond formation as described in Procedure F. The reaction mixture was subjected directly to purification by semi-preparative RP-HPLC (5 – 50% AcN in H_2_O with 0.1% (v/v) TFA over 40 min) yielding the desired product as a white solid (25 mg, isolated yield: 22%, >95% purity).

**Analytical RP-HPLC R_t_** (5 – 95% AcN in H_2_O with 0.1% (v/v) TFA over 20 min, 220 nm and 254 nm): 10.223 min.

**HRMS** (*m/z* ESI^+^): calculated for C_50_H_74_N_15_O_12_S_2_ (M+H)^+1^= 1140.5083; observed 1140.5058, calculated for C_50_H_75_N_15_O_12_S_2_ (M+2H)^+2^= 570.7580; observed 570.7584.

**
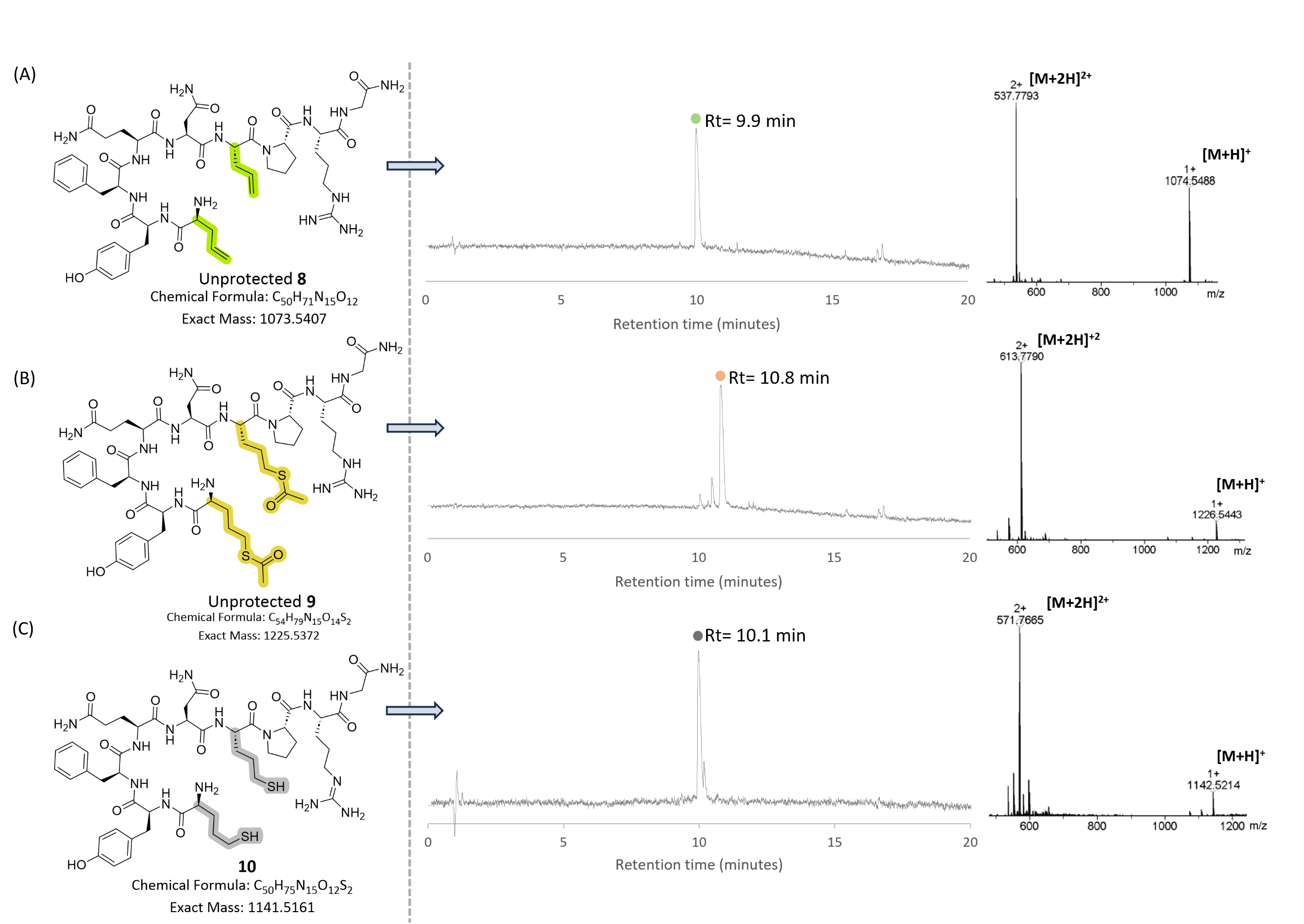
**

**Figure S1**. (**A**) Analytical RP-HPLC (220 nm) trace of the unprotected starting material **8**. ESI-MS analysis of the main peak with a retention time of 9.9 minutes confirmed the mass of the unprotected peptide **8** (calculated for C_50_H_72_N_15_O_12_ (M+H)^+1^ =1074.5485; observed 1074.5488, calculated for C_50_H_73_N_15_O_12_ (M+2H)^+2^ = 537.7793; observed 537.7793). (**B**) Analytical RP-HPLC (220 nm) trace of the unprotected peptide **9**. ESI-MS analysis of the main peak with a retention time of 10.8 minutes confirmed the mass of the unprotected peptide **9** (calculated for C_54_H_80_N_15_O_14_S_2_ (M+H)^+1^ = 1226.5451; observed 1226.5443, calculated for C_54_H_81_N_15_O_14_S_2_ (M+2H)^+2^ = 613.7764; observed 613.7790). (**C**) Analytical RP-HPLC (220 nm) trace of the peptide **10**. ESI-MS analysis of the main peak with a retention time of 10.1 minutes confirmed the mass of the peptide **10** (calculated for C_50_H_76_N_15_O_12_S_2_ (M+H)^+1^= 1142.5239; observed 1142.5214, calculated for C_50_H_77_N_15_O_12_S_2_ (M+H)^+1^= 571.7659; observed 571.7665).

**SST Analog 1**

The peptide was synthesized at 0.1 mmol scale by automated SPPS as described in Procedure B using Fmoc-Cys(Trt)-OH, Fmoc-Ser(*t*Bu)-OH, Fmoc-Thr(*t*Bu)-OH, Fmoc-Phe-OH, Fmoc-Thr(*t*Bu)-OH, Fmoc-Lys(Boc)-OH, Fmoc-Trp(Boc)-OH, Fmoc-Phe-OH, Fmoc-Phe-OH, Fmoc-Asn(Trt)-OH, Fmoc-Lys(Boc)-OH, Fmoc-AgI-OH, Fmoc-Gly-OH, Boc-Ala-OH. ATE and *S*-deacetylation reactions were carried out on-resin as described in Procedures C and D, respectively. Resin cleavage and global deprotection of the peptide were carried out as described in Procedure E. The linear peptide precursor was cyclized *via* disulfide formation as described in Procedure F. The reaction mixture was subjected directly to purification by semi-preparative RP-HPLC (5 – 50% AcN in H_2_O with 0.1% (v/v) TFA over 40 min) yielding the desired product as a white solid (23 mg, isolated yield: 14%, >95% purity).

**Analytical RP-HPLC R_t_** (5 – 95% AcN in H_2_O with 0.1% (v/v) TFA over 20 min, 220 nm and 254 nm): 11.568 min.

**HRMS** (*m/z* ESI^+^): calculated for C_78_H_111_N_19_O_18_S_2_ (M+2H)^+2^= 832.8859; observed 832.8896, calculated for C_78_H_112_N_19_O_18_S_2_ (M+3H)^+3^= 555.9353; observed 555.9337.

**SST Analog 2**

The peptide was synthesized at 0.10 mmol scale by automated SPPS as described in Procedure B using Fmoc-AgI-OH, Fmoc-Ser(*t*Bu)-OH, Fmoc-Thr(*t*Bu)-OH, Fmoc-Phe-OH, Fmoc-Thr(*t*Bu)-OH, Fmoc-Lys(Boc)-OH, Fmoc-Trp(Boc)-OH, Fmoc-Phe-OH, Fmoc-Phe-OH, Fmoc-Asn(Trt)-OH, Fmoc-Lys(Boc)-OH, Fmoc-AgI-OH, Fmoc-Gly-OH and Boc-Ala-OH. ATE and *S*-deacetylation reactions were carried out on-resin as described in Procedures C and D, respectively. Resin cleavage and global deprotection of the peptide were carried out as described in Procedure E. The linear peptide precursor was cyclised *via* disulfide formation as described in Procedure F. The reaction mixture was subjected directly to purification by semi-preparative RP-HPLC (5 – 50% AcN in H_2_O with 0.1% (v/v) TFA over 40 min) yielding the desired product as a white solid (20 mg, isolated yield: 12%, >95% purity).

**Analytical RP-HPLC R_t_** (5 – 95% AcN in H_2_O with 0.1% (v/v) TFA over 20 min, 220 nm and 254 nm): 11.769 min.

**HRMS** (*m/z* ESI^+^): calculated for C_80_H_115_N_19_O_18_S_2_ (M+2H)^+2^= 846.9054; observed 846.9042, calculated for C_80_H_116_N_19_O_18_S_2_ (M+3H)^+3^= 564.9395; observed 564.9422.

**tert-butyl (3-aminopropyl)carbamate (14)**

To a stirring solution of 1,3-diaminopropane (**13**, 9.6 mL, 114.55 mmol) in CHCl_3_ (100 mL) was added slowly over 3 h a solution of Boc_2_O (5 g, 22.9 mmol) in CHCl_3_ (50 mL) at 0°C. The reaction mixture was stirred at rt for 18 h and the solvent was removed under reduced pressure. The white crude solid was dispersed in H_2_O (130 mL), extracted with CH_2_Cl_2_ (3 × 100 mL) and the organic layers were combined, washed with brine (2 × 50 mL), H_2_O (2 × 50 mL) and removed under reduced pressure*.* The crude product was purified by silica gel flash chromatography (CHCl_3_:MeOH– 2:1) to give a pale-yellow oil (3.5 g, 88%). The compound characterization was in good agreement with the literature.^49^

**^1^H-NMR** (400 MHz, CDCl_3_) δ_Η_ 5.09 (bs, CONH), 3.21 – 3.08 (m, 2H, CONHCH_2_), 2.72 (t, *J =* 6.7 Hz, 2H, CH_2_NH_2_), 1.80 (bs, 2H, NH_2_), 1.57 (app.p, 2H, CONHCH_2_CH_2_), 1.39 (s, 9H, C(CH_3_)_3_).

**^13^C NMR** (151 MHz, DMSO-*d_6_*) δ_C_ 156.1 (Boc C=O), 79.1 (C(CH_3_)_3_), 39.5 (NH_2_CH_2_), 38.2 (NH_2_CH_2_CH_2_CH_2_), 33.2 (NH_2_CH_2_CH_2_), 28.4 (C(CH_3_)_3_).

**HRMS** (*m/z* APCI^+^): calculated for C_8_H_19_N_2_O_2_ (M+H)^+1^= 175.1447; observed 175.1442.

***tert*-butyl (3-(2,5-dioxo-2,5-dihydro-1H-pyrrol-1-yl)propyl)carbamate (15)**

To a stirred solution of **14** (3.16 g, 18.14 mmol) in anhydrous toluene (50 mL) was added dropwise a solution of maleic anhydride (2.13 g, 21.76 mmol) in anhydrous toluene (10 mL) and the reaction mixture was stirred at rt for 18 h. The solvent was removed under reduced pressure to give a white crude solid which was subsequently cyclized without further purification. The crude acid was dissolved in Ac_2_O (18 mL) and sodium acetate (1.49 g, 18.14 mmol) was added portionwise to the solution. The reaction was stirred at 70 °C under Ar for 18 h. The solvent was removed under reduced pressure and crude product was purified by silica gel flash chromatography (ΕtΟAc:Hex– 13:7) yielding a colorless oil (1.61 g, 35% over two steps). The compound characterization was in good agreement with the literature.^50^

**^1^H-NMR** (400 MHz, CDCl_3_) δ_Η_ 6.70 (bs, 2H, CH=CH) 4.95 (bs, 1H, NH), 3.58 (t, *J =* 6.6 Hz, 2H, NHCH_2_CH_2_CH_2_), 3.08 (dd, *J =* 12.2, 6.6 Hz, 2H, NHCH_2_), 1.75 (app.p, 2H, NHCH_2_CH_2_), 1.43 (s, 9H, C(CH_3_)_3_).

**^13^C NMR** (151 MHz, DMSO-*d_6_*) δ_C_ 171.2 (Mal C=O), 155.9 (Boc C=O), 134 (CH=CH), 79.2 (C(CH_3_)_3_), 37.4 (NHCH_2_CH_2_CH_2_), 35.1 (NHCH_2_), 28.8 (NHCH_2_CH_2_), 28.4 (C(CH_3_)_3_).

**HRMS** (*m/z* APCI^-^): calculated for C_12_H_17_N_2_O_4_ (M-H)^-1^= 253.1267; observed 253.1273.

**3-(2,5-dioxo-2,5-dihydro-1H-pyrrol-1-yl)propan-1-aminium 2,2,2-trifluoroacetate (16)**

TFA (10 mL) was added dropwise to a stirring mixture of **15** (1.73 g, 6.00 mmol) in CH_2_Cl_2_ (30 mL) and the reaction was stirred at rt for 2 h. The mixture was then diluted with CH_2_Cl_2_ (20 mL) and H_2_O (20 mL) and extracted with H_2_O (3 × 30 mL). The combined aqueous layers were washed with CH_2_Cl_2_ (3 × 30 mL) and concentrated under reduced pressure yielding a pure white solid (1.73 g, 99%). The compound characterization was in good agreement with the literature.^51^

**^1^H-NMR** (400 MHz, CDCl_3_) δ_Η_ 7.85 (bs, 3H, NH_3_^+^) 7.03 (s, 2H, CH=CH), 3.46 (t, *J =* 6.9 Hz, 2H, NH_3_CH_2_CH_2_CH_2_), 2.87 – 2.67 (m, 2H, NH_3_CH_2_), 1.85 – 1.70 (m, 2H, NH_3_CH_2_CH_2_).

**^13^C NMR** (151 MHz, DMSO-*d_6_*) δ_C_ 171.1 (Mal C=O), 134.5 (CH=CH), 36.6 (NH_3_CH_2_), 34.4 (NH_3_CH_2_CH_2_CH_2_), 26.4 (NH_3_CH_2_CH_2_).

**HRMS** (*m/z* APCI^+^): calcd. for C_7_H_11_N_2_O_2_ (M+H)^+1^= 155.0821; observed 155.0807.

**tert-butyl(2-((3-(2,5-dioxo-2,5-dihydro-1H-pyrrol-1-yl)propyl)carbamoyl)phenyl)carbamate (17)**

A solution of Boc-Abz-OH (1.0 g, 3.73 mmol) in CH_2_Cl_2_ (10 mL) was added dropwise to a stirring solution of **15** (0.8 g, 3.73 mmol), EDC^.^HCl (1.44 g, 7.46 mmol) and TEA (2.0 mL, 14.91 mmol) in CH_2_Cl_2_ (40 mL) at 0 °C and the reaction was stirred at rt for 18 h. The solution was then washed with brine (3 × 30 mL), H_2_O (3 × 30 mL), dried over MgSO_4_ and the solvent was removed under reduced pressure*.* The crude product was purified by silica gel flash chromatography (ΕtΟAc:Hex– 8:2) to give a pale yellow oil (0.96 g, 69%).

**^1^H-NMR** (600 MHz, DMSO-*d_6_*) δ_Η_ 10.35 (s, 1H, Boc NH), 8.38 (d, *J =* 7.9 Hz, 1H, Abz ArCH), 7.58 (dd, *J =* 7.9, 1.4 Hz, 1H, Abz ArCH), 7.47 – 7.42 (m, 1H, Abz ArCH), 7.05 – 7.01 (m, 1H, Abz ArCH), 6.98 (s, 1H, NHCH_2_CH_2_), 6.75 (s, 2H, CH=CH), 3.70 – 3.63 (m, 2H, NHCH_2_CH_2_CH_2_), 3.38 (dd, *J =* 12.4, 6.2 Hz, 2H, NHCH_2_CH_2_CH_2_), 1.93 – 1.84 (m, 2H, NHCH_2_CH_2_CH_2_), 1.51 (s, 9H, C(CH_3_)_3_).

**^13^C NMR** (151 MHz, DMSO-*d_6_*) δ_C_ 171.2 (Mal C=O), 168.9 (Abz C=O), 153.1 (Boc C=O), 140.6 (Abz qC), 134.3 (CH=CH), 132.5 (Abz Ar C), 126.5 (Abz Ar C), 121.4 (Abz Ar C), 119.8 (Abz Ar C), 119.2 (Abz qC), 80.2 (C(CH_3_)_3_), 36.1 (NHCH_2_), 34.7 (NHCH_2_CH_2_CH_2_), 28.7 (C(CH_3_)_3_), 28.0 (NHCH_2_CH_2_).

**HRMS** (*m/z* APCI^-^): calculated for C_19_H_22_N_3_O_5_ (M-H)^-1^= 372.1559; observed 372.1571.

**ν_max_** (film)/cm^-1^: 3343 (N-H), 3086 (C-H), 2980 (CH_2_), 1704 (C=O), 1690 (C=C), 1431 (Ar C-C).

**RGD peptide 18**

The peptide **12** was synthesized at 0.10 mmol scale by automated SPPS as described in Procedure B using Fmoc-Gly-OH, Fmoc-Glu(O*t*Bu)-OH, Fmoc-Thr(*t*Bu)-OH, Fmoc-Phe-OH, Fmoc-Val-OH, Fmoc-Asp(O*t*Bu)-OH, Fmoc-Gly-OH, Fmoc-Arg(Pbf)-OH, Fmoc-Thr(*t*Bu)-OH, Fmoc-Val-OH, Fmoc-Gln(Trt)-OH, Fmoc-Pro-OH, Boc-Agl-OH. ATE and *S*-deacetylation reactions were carried out on-resin as described in Procedures C and D, respectively. The fluorophore **17** was covalently attached at the *N*-terminus of the linear RGD peptide by preparing a solution of **17** (10 equiv.) and TEA (10 equiv.) in DMF (3 mL) and adding it to the resin for 1 h. Resin cleavage and global deprotection of the peptide were carried out as described in Procedure E, yielding the desired conjugated product **18** in high purity as confirmed by analytical RP-HPLC.

**Analytical RP-HPLC R_t_** (5 – 95% AcN in H_2_O with 0.1% (v/v) TFA over 30 min, 220nm): 10.983 min.

**HRMS** (*m/z* ESI^+^): calculated for C_75_H_115_N_21_O_23_S (M+2H)^+2^= 854.9097; observed 8

54.9065, calculated for C_75_H_116_N_21_O_23_S (M+3H)^+3^= 570.2758; observed 570.2753.

# NMR Spectra and Analytical PR-HPLC Traces.

**Tetrapeptide 1**

**
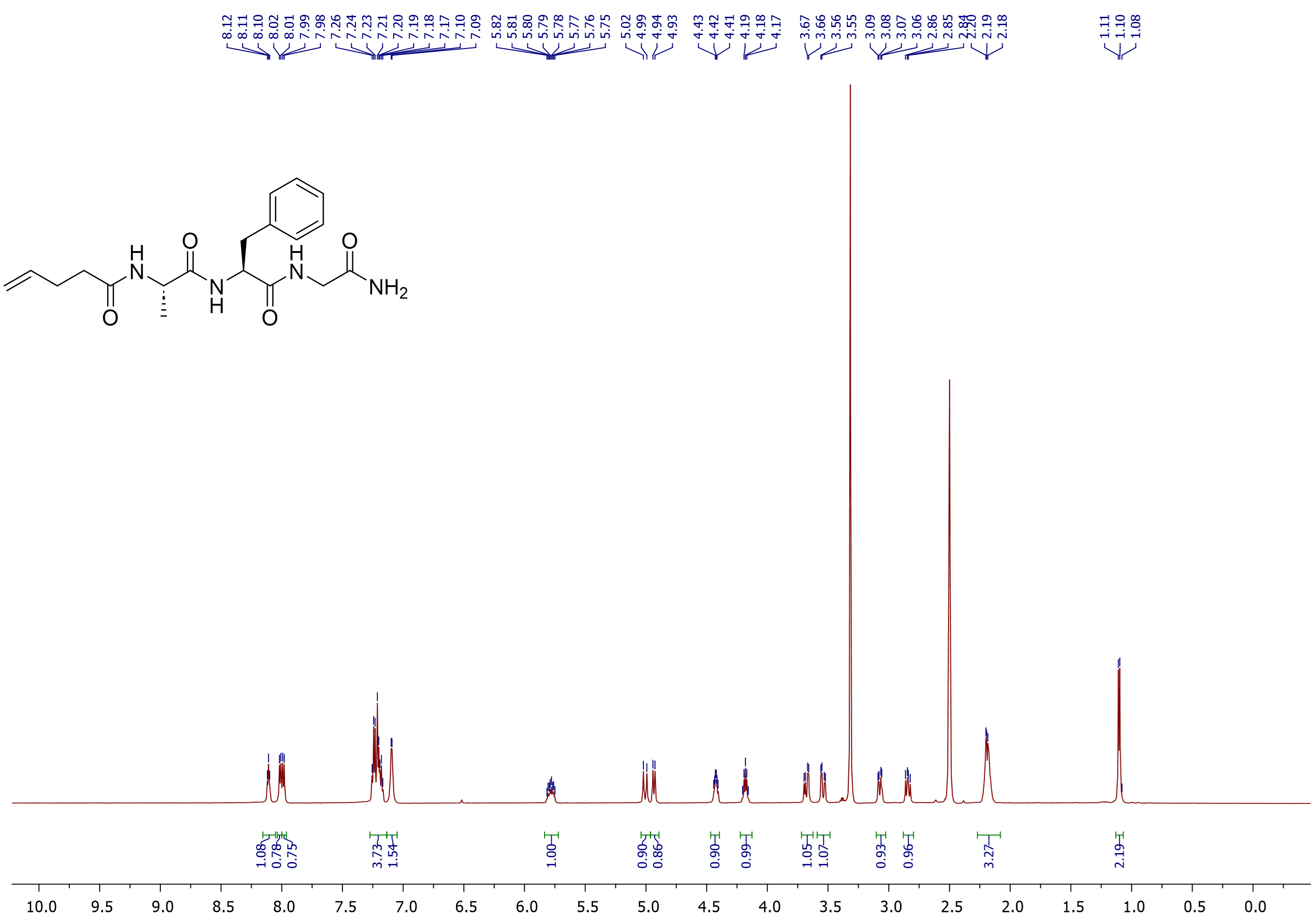
**

**Figure S2**. ^1^H NMR spectrum of the tetrapeptide **1**.

**Figure S3**. ^13^C NMR spectrum of the tetrapeptide **1**.

**Tetrapeptide 2**

**
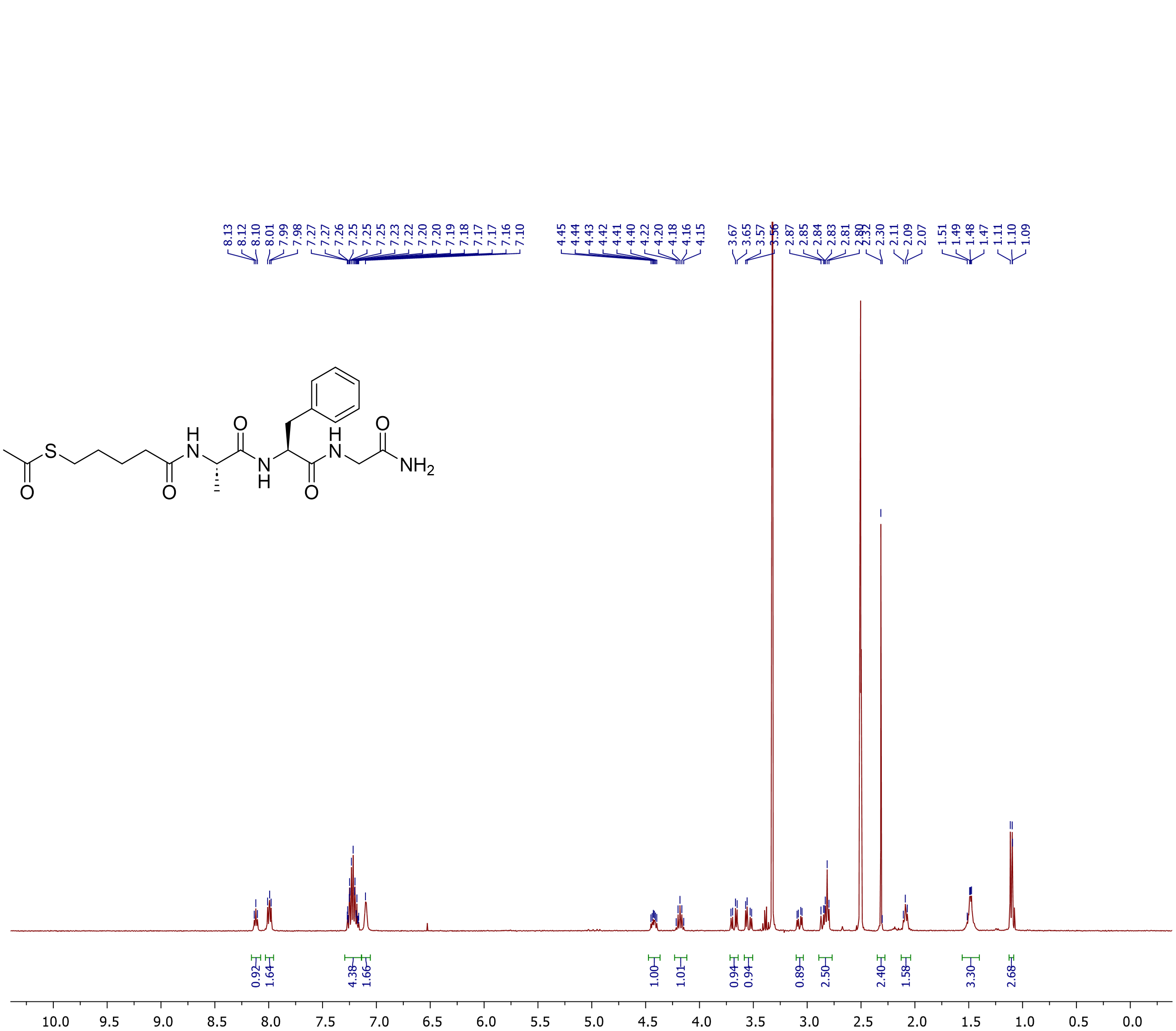
**

**Figure S4**. ^1^H NMR spectrum of the tetrapeptide **2**.

**Figure S5**. ^13^C NMR spectrum of the tetrapeptide **2**.

**Tetrapeptide 4**

**
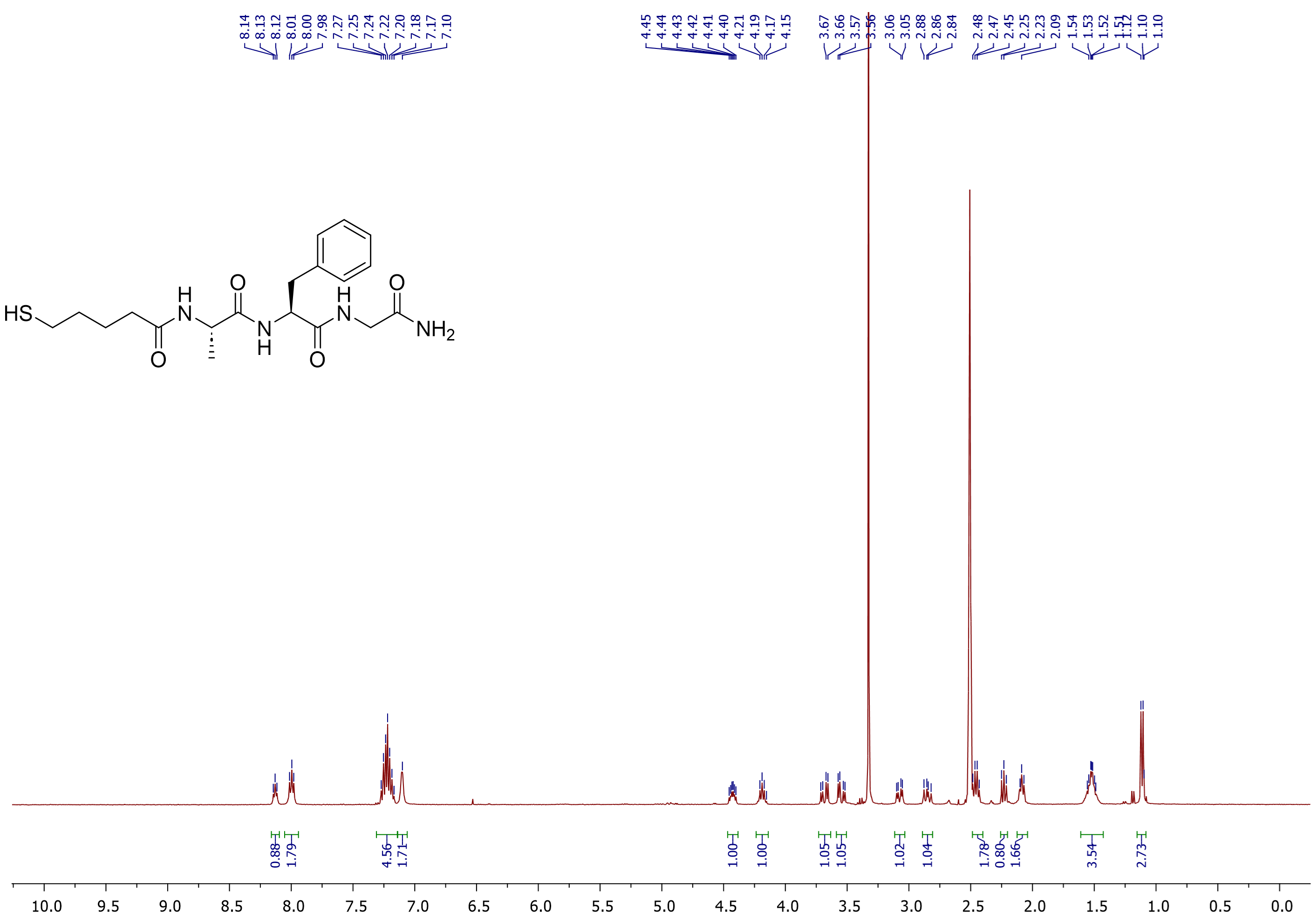
**

**Figure S6**. ^1^H NMR spectrum of the tetrapeptide **4**.

**Figure S7**. ^13^C NMR spectrum of the tetrapeptide **4**.

**Fluorophore 17**


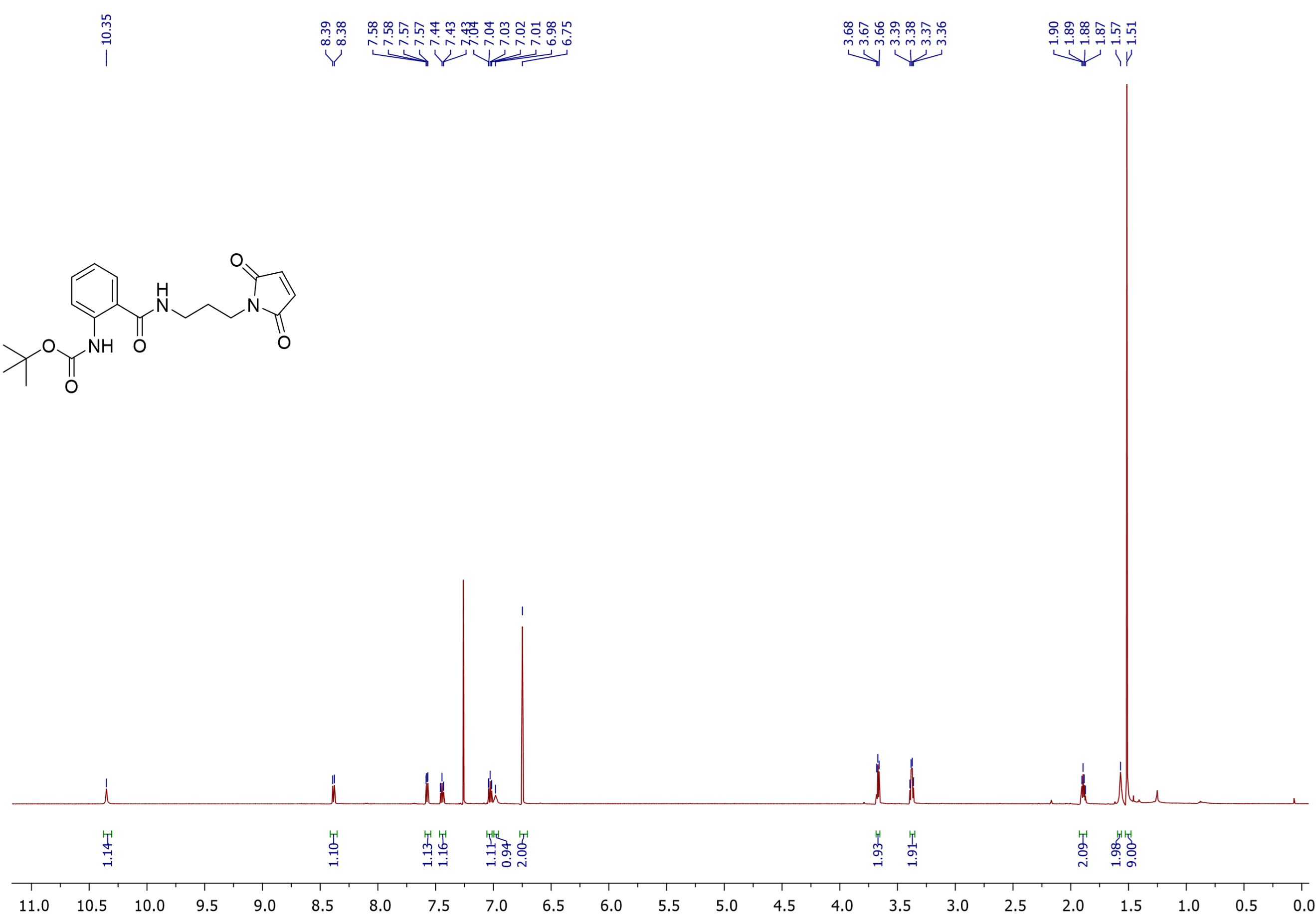


**Figure S8**. ^1^H NMR spectrum of **17**.

**Figure S9**. ^13^C NMR spectrum of **17**.

**VP Analog 1**


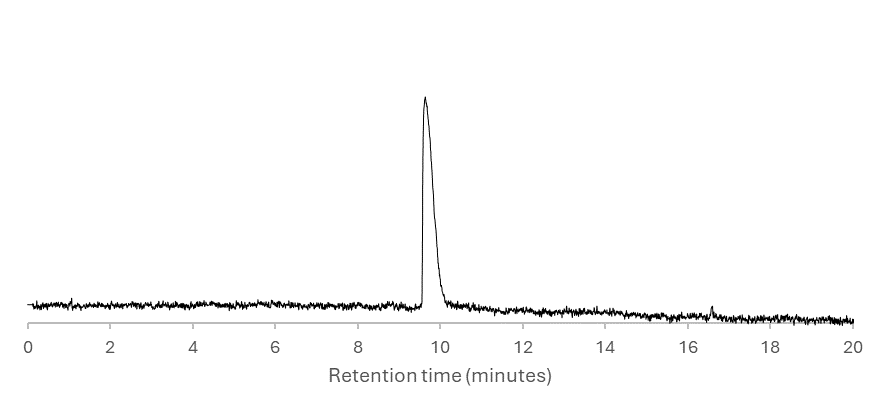


**Figure S10**. Analytical RP-HPLC trace (220 nm) of the pure VP analog **1** (R_t_= 9.827 minutes).

**
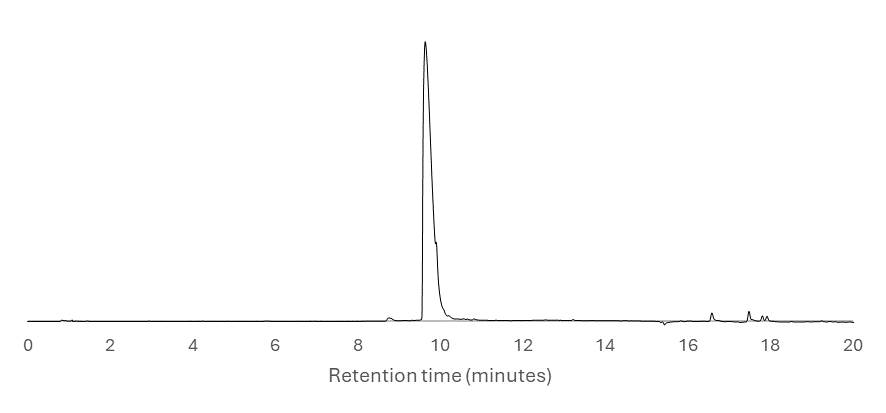
**

**Figure S11**. Analytical RP-HPLC trace (254 nm) of the pure VP analog **1** (R_t_= 9.827 minutes).

**
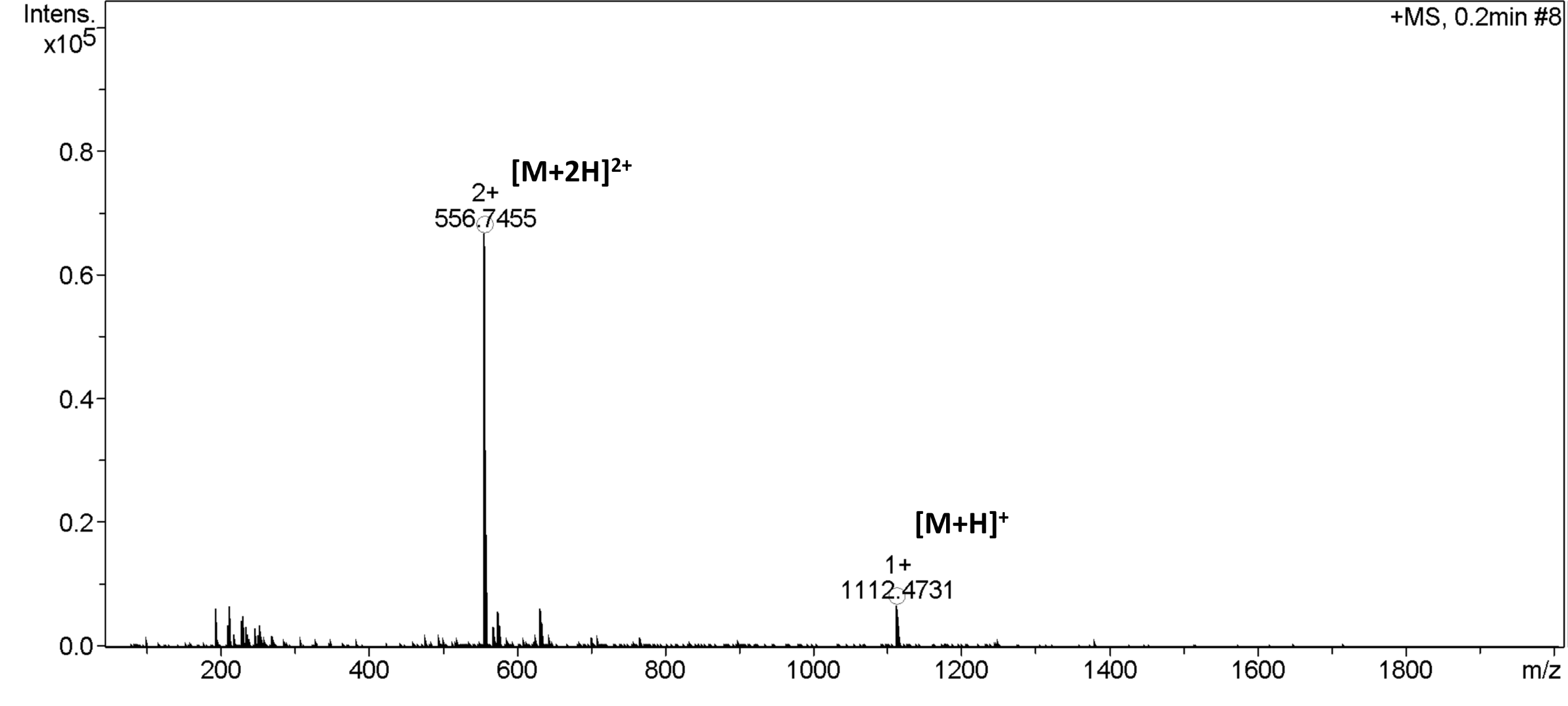
 Figure S12**. ESI-MS of the pure VP analog **1**: calculated for C_48_H_70_N_15_O_12_S_2_ (M+H)^+1^= 1112.4770; observed 1112.4731, calculated for C_48_H_71_N_15_O_12_S_2_ (M+2H)^+2^ = 556.7424; observed 556.7455.

**VP Analog 2**


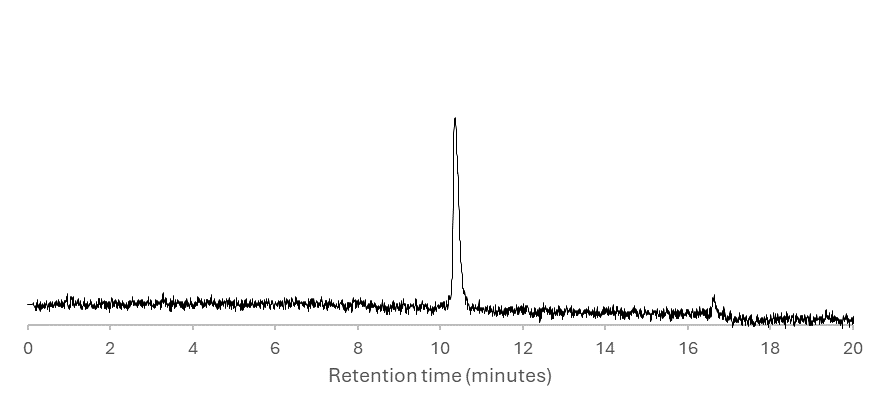


**Figure S13**. Analytical RP-HPLC trace (220 nm) of the pure VP analog **2** (R_t_= 10.223 minutes).


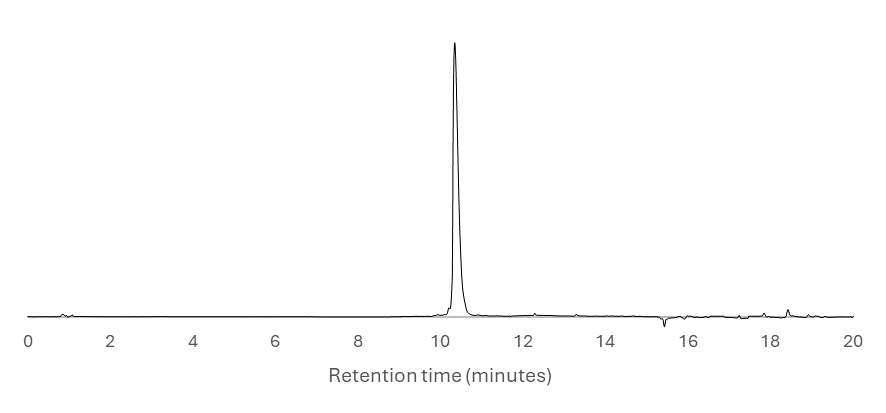


**Figure S14**. Analytical RP-HPLC trace (254 nm) of the pure VP analog **2** (R_t_= 10.223 minutes).


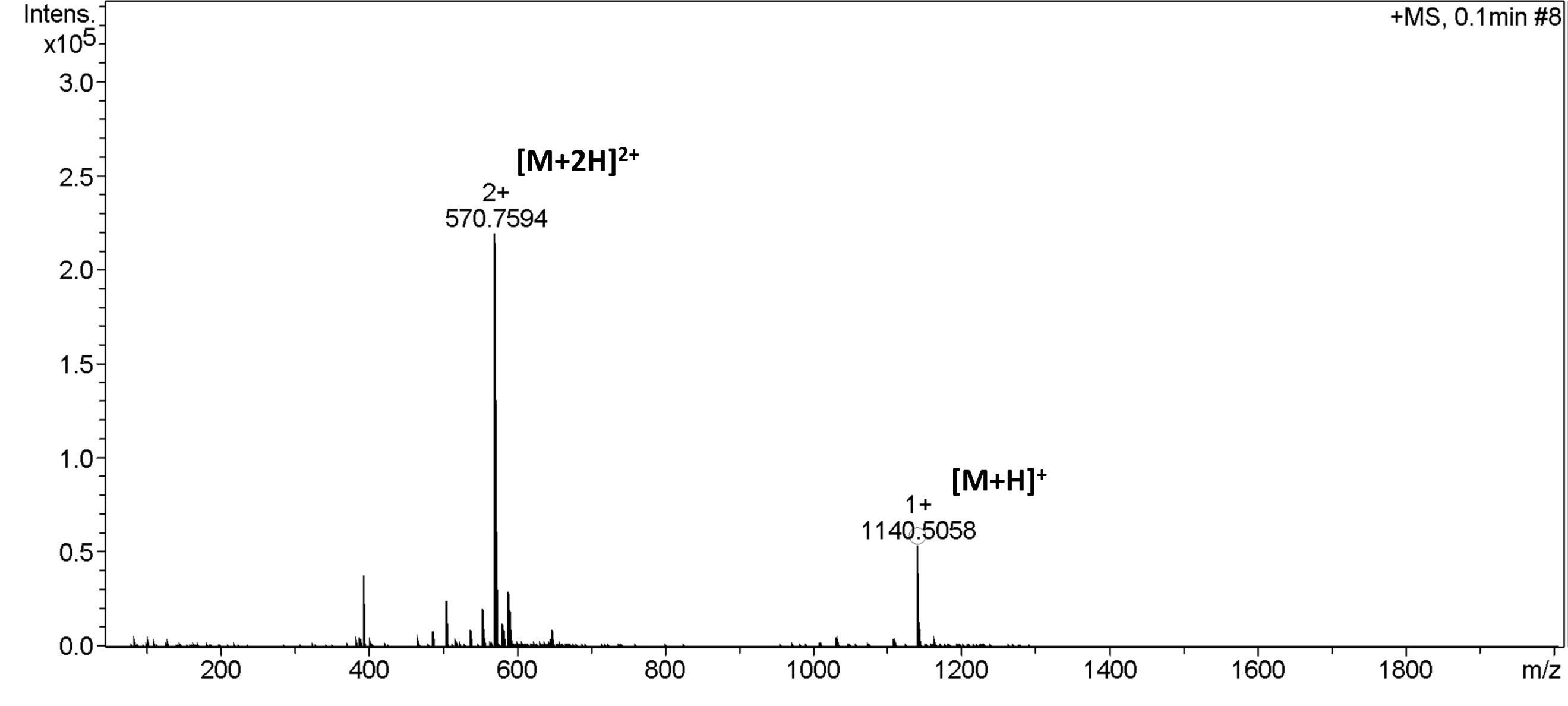


**Figure S15**. ESI-MS of the pure VP analog **2**: calculated for C_50_H_74_N_15_O_12_S_2_ (M+H)^+1^= 1140.5083; observed 1140.5058, calculated for C_50_H_75_N_15_O_12_S_2_ (M+2H)^+2^= 570.7580; observed 570.7584.

**SST Analog 1**


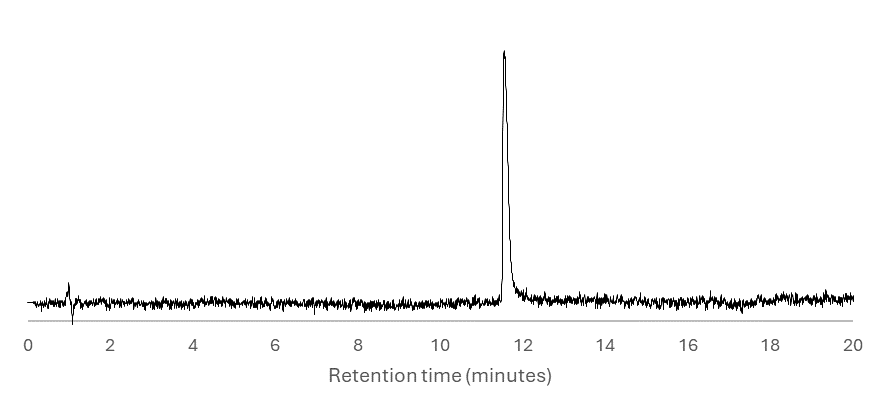


**Figure S16**. Analytical RP-HPLC trace (220 nm) of the pure SST analog **1** (R_t_= 11.568 minutes).

**
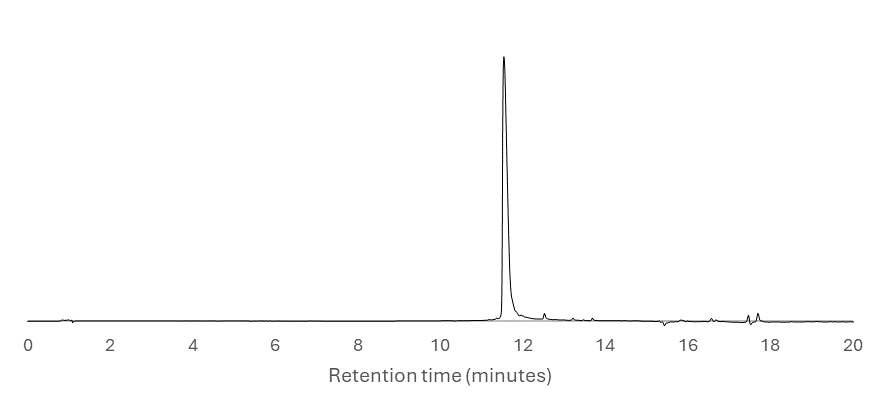
**

**Figure S17**. Analytical RP-HPLC trace (254 nm) of the pure SST analog **1** (R_t_= 11.568 minutes).


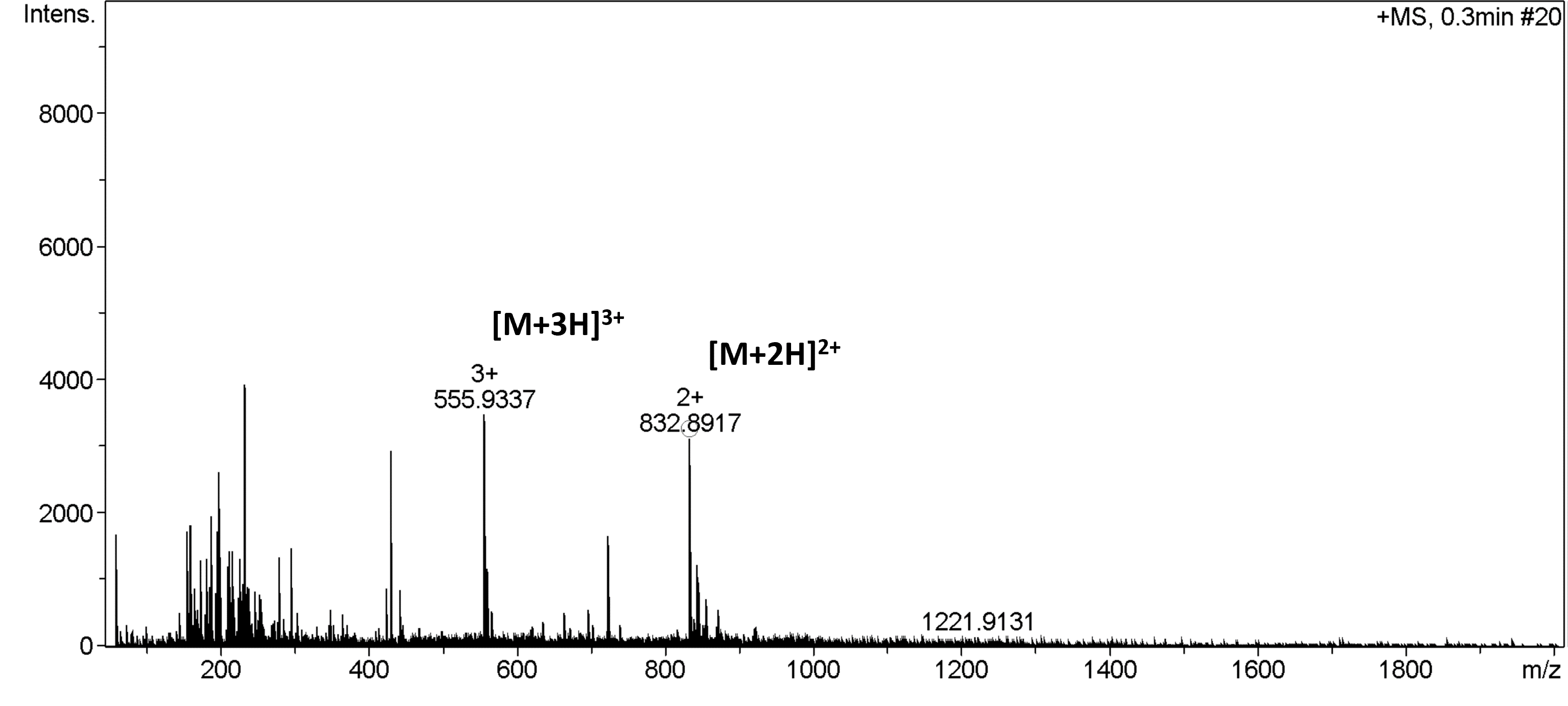
**Figure S18**. ESI-MS of the pure SST analog **1**: calculated for C_78_H_111_N_19_O_18_S_2_ (M+2H)^+2^= 832.8919; observed 832.8917, calculated for C_78_H_112_N_19_O_18_S_2_ (M+3H)^+3^= 555.9353; observed 555.9337.

**SST Analog 2**


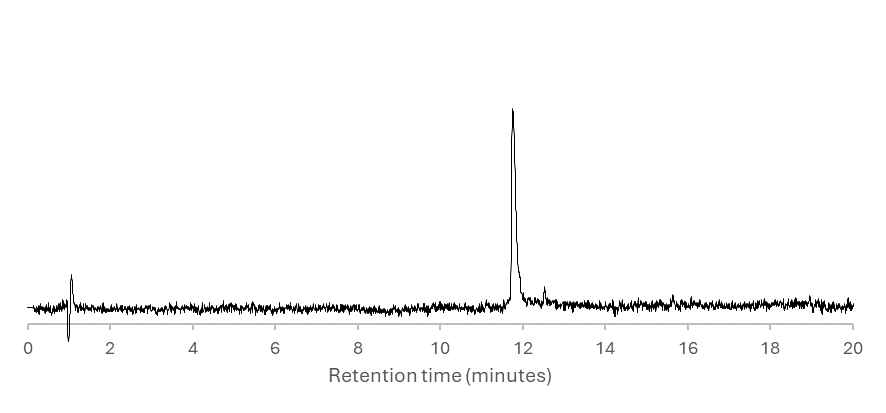


**Figure S19**. Analytical RP-HPLC trace (220 nm) of the pure SST analog **2** (R_t_= 11.769 minutes).


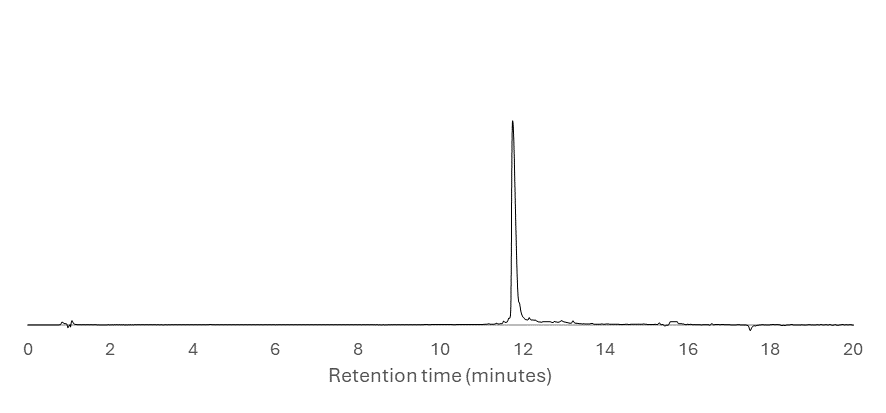


**Figure S20**. Analytical RP-HPLC trace (254 nm) of the pure SST analog **2** (R_t_= 11.769 minutes).


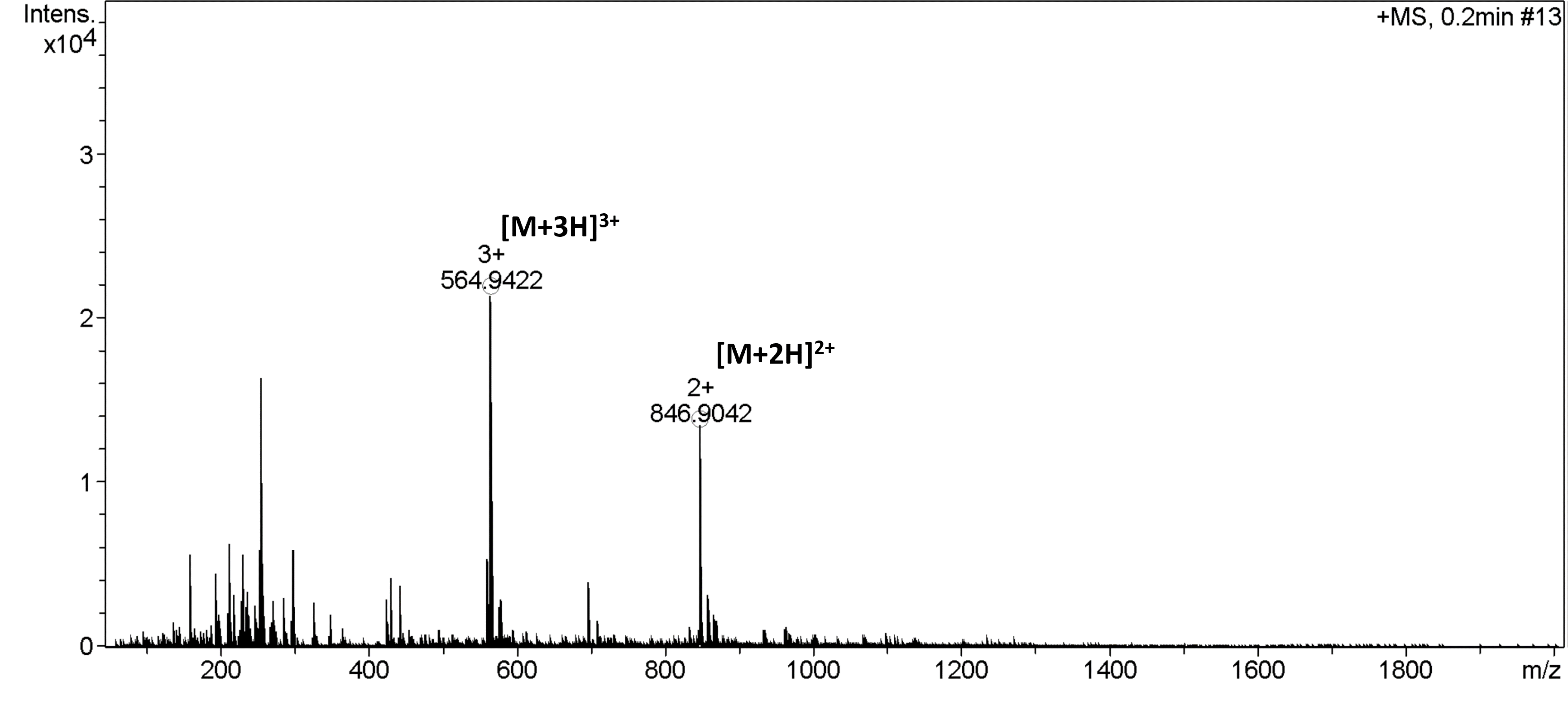


**Figure S21**. ESI-MS of the pure SST analog **2**: found: calculated for C_80_H_115_N_19_O_18_S_2_ (M+2H)^+2^= 846.9054; observed 846.9042, calculated for C_80_H_116_N_19_O_18_S_2_ (M+3H)^+3^= 564.9395; observed 564.9422.

**RGD peptide 18**

**Figure S22**. Analytical RP-HPLC trace (220 nm) of the crude linear RGD peptide **18** bearing the Abz fluorophore at the *N*-terminus of the sequence (R_t_= 10.983 minutes).

**
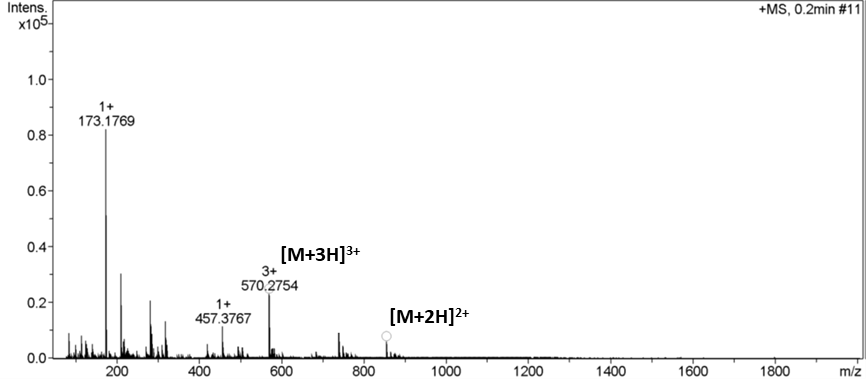
**

**Figure S23**. ESI-MS of the crude linear RGD **18**: calculated for C_75_H_115_N_21_O_23_S (M+2H)^+2^= 854.9097; observed 854.9065, calculated for C_75_H_116_N_21_O_23_S (M+3H)^+3^= 570.2758; observed 570.2753.

## References

1. L. Nørskov-Lauritsen, A.R.B. Thomsen, H. Bräuner-Osborne, *Int. J. Mol. Sci.* **2014**, *15*, 2554-2572.
2. K. Dabrowa, M. Pawlak, P. Duszewski, J. Jurczak, *Org. Lett.* **2012**, *14*, 6298–6301.
3. D. Kalia, P. V. Malekar, M. Parthasarathy*, Angew. Chem. Int. Ed*, **2016**, *55*, 432–1435.
4. L.D. Turner, A.L. Nielsen, L. Lin, S. Pellett, T. Sugane, M.E. Olson, E.A. Johnson and K.D. Janda, *RSC Med. Chem*., 2021, **12**, 960–969.
